# Spatiotemporal Analysis of West Nile Virus Epidemic in South Banat District, Serbia, 2017–2019

**DOI:** 10.3390/ani11102951

**Published:** 2021-10-13

**Authors:** Sonja Radojicic, Aleksandar Zivulj, Tamas Petrovic, Jakov Nisavic, Vesna Milicevic, Sandra Sipetic-Grujicic, Dusan Misic, Malgorzata Korzeniowska, Slavoljub Stanojevic

**Affiliations:** 1 Department of Infectious Animal Diseases and Diseases of Bees, Faculty of Veterinary Medicine, University of Belgrade, Bulevar Oslobođenja 18, 11000 Belgrade, Serbia; sonjar@vet.bg.ac.rs; 2 Veterinary Specialist Institute Pancevo, Novoseljanski Put 33, 13000 Pancevo, Serbia; acazivulj@vsipancevo.co.rs; 3 Scientific Veterinary Institute Novi Sad, Rumenacki Put 20, 21000 Novi Sad, Serbia; tomy@niv.ns.ac.rs; 4 Department of Microbiology, Faculty of Veterinary Medicine, University of Belgrade, Bulevar Oslobođenja 18, 11000 Belgrade, Serbia; jakovmoni@vet.bg.ac.rs; 5 Scientific Veterinary Institute of Serbia, Janisa Janulisa 14 Street, 11107 Belgrade, Serbia; vesna.milicevic@nivs.rs; 6 Faculty of Medicine, Institute of Epidemiology, Belgrade University, 11000 Belgrade, Serbia; sandra.grujicic@mfub.bg.ac.rs; 7 Department of Functional Foods Development, Wroclaw University of Environmental and Life Sciences, Chelmonskiego Street 37, 51-630 Wroclaw, Poland; dusan.misic@upwr.edu.pl (D.M.); malgorzata.korzeniowska@upwr.edu.pl (M.K.); 8 Directorate of National Reference Laboratories, Batajnicki Drum 10, 11080 Zemun, Serbia

**Keywords:** West Nile virus, mosquitoes, sentinel animals, spatial analysis, GIS

## Abstract

**Simple Summary:**

West Nile fever is an arthropod-borne viral disease that is transmitted from birds to humans and animals by mosquitoes. Humans may develop a severe disease, which sometimes can be fatal. At the end of the 20th century, the first outbreaks of West Nile fever among humans in urban environments in Eastern Europe and the United States were reported. The epidemics were characterized by the neurological form of the disease with a fatal outcome. Since the first outbreak of the disease in Serbia, the highest number of cases occurred in 2018. West Nile fever spread is driven by location and time, which means nearby locations and periods have similar features. Recognition of patterns of spread of the disease has the potential to facilitate the mosquito control program and disease prevention. This study aimed to examine the geographical and temporal similarities of registered cases during the epidemics in the period 2017–2019 in South Banat District, Serbia. We identified the following factors as crucial for the prediction of possible outbreaks: the presence of virus in natural reservoirs, mosquito abundance; precipitation, high water level of rivers followed by a consequent sudden decrease of precipitation and withdrawal of rivers into the main bed, and favorable temperatures.

**Abstract:**

West Nile virus (WNV) is an arthropod-born pathogen, which is transmitted from wild birds through mosquitoes to humans and animals. At the end of the 20th century, the first West Nile fever (WNF) outbreaks among humans in urban environments in Eastern Europe and the United States were reported. The disease continued to spread to other parts of the continents. In Serbia, the largest number of WNV-infected people was recorded in 2018. This research used spatial statistics to identify clusters of WNV infection in humans and animals in South Banat County, Serbia. The occurrence of WNV infection and risk factors were analyzed using a negative binomial regression model. Our research indicated that climatic factors were the main determinant of WNV distribution and were predictors of endemicity. Precipitation and water levels of rivers had an important influence on mosquito abundance and affected the habitats of wild birds, which are important for maintaining the virus in nature. We found that the maximum temperature of the warmest part of the year and the annual temperature range; and hydrographic variables, e.g., the presence of rivers and water streams were the best environmental predictors of WNF outbreaks in South Banat County.

## 1. Introduction

West Nile virus (WNV) is a single-stranded RNA arbovirus from the Flaviviridae family, genus Flavivirus. WNV is primarily a zoonotic agent transmitted between birds as the main reservoir hosts and mosquitoes as vectors. Humans and horses can be infected as spillovers host, but the infection in these organisms ends without further transmission of the virus, i.e., these organisms are dead-end hosts [1,2]. WNV is today considered the most important cause of viral encephalitis in humans worldwide [3].

The WNV has been isolated or identified serologically in many vertebrate species [4]. Since 1998, cases of viral encephalitis caused by WNV in horses have been reported in Italy, France, and North America. Research conducted in parts of Europe and the Middle East has established that as much as one-third of the tested horses were exposed to the WNV, with or without symptoms of the disease [5,6].

Since the discovery of the first infection in humans in 1937 [4], there has been a significant spread of the disease globally. In most cases, the infection was asymptomatic and only a few cases of severe neurological forms of the disease were reported [2]. At the end of the 20th century, the first West Nile fever WNF outbreaks among humans in urban environments in Eastern Europe and the United States were reported [2]. Since 2012, human WNF cases have been recorded every year in Serbia. In terms of the number of patients, the years 2013 and 2018 stand out, when the largest numbers of clinical cases of infection were registered [7]. The epidemics were characterized by a serious neurological form of the disease with a fatal outcome. During these epidemics, several common characteristics were observed at the time of the disease outbreaks: the *Culex pipiens* mosquito was identified as a vector of the disease, significantly less precipitation was registered than the usual multi-year average, significantly higher than normal summer temperatures were recorded, and all areas were located near large rivers which provide adequate living conditions for residential and migratory species of wild birds [2]. Besides, the epidemic in the northeastern United States was accompanied by the epizootic in birds, especially crows [8]. The sudden appearance of WNF in the United States can also be related to changes in the feeding habits of *Cx. pipiens*, the dominant enzootic species. Shifts in feeding habits from competent avian hosts early in the early stage of the epidemic to incompetent humans after mosquito infection, resulted in synergistic effects that greatly amplified the number of human infections [9].

Given that WNF is an arthropod-borne disease, the spread of the disease is conditioned by climatic variations and landscape changes to the natural habitats of the mosquitoes and available bird habitats [1,4], geospatial data can be used for risk prediction and risk mapping [10]. With the development of geographic information system (GIS) software and other related tools for spatial data analysis, substantial progress has been made in risk analysis [11]. Numerous studies have used geospatial data and GIS software for detecting, analyzing, and predicting spatial patterns of disease occurrence [10,12,13,14]. The advantage of the GIS analytical tool is the ability to integrate and analyze risk factors and create WNV risk maps for humans and other susceptible hosts [15]. In addition to visualizing epidemiological data and presenting the geographical distribution of the disease in a much more predictive manner, the spatiotemporal analysis identifies spatial and temporal clusters and identifies risk geographic areas. Spatial inquiry can also inform vector control policy, including where to prioritize limited control resources [16,17,18]. By identifying locations of the WNF hot spots, the mosquito control treatments can be rationally and more efficiently applied primarily in high-risk areas and, therefore, will more efficiently decrease virus transmission [17,19].

The analyses conducted in this paper aimed to discover spatial, temporal, and spatiotemporal clusters of WNV infection in humans and animals in South Banat District, Serbia, also researching for causal relationships, i.e., identifying such clusters of WNV cases and researching possible causal or associative relationships with meteorological factors. Our focus was on understanding spatiotemporal patterns and risk factors associated with WNV infection in humans and domestic animals. An additional aim was to investigate the epidemiological characteristics of WNV outbreaks and provide a scientific basis for the effective control of this disease. Spatial research into the geography of the West Nile disease in Serbia includes WNV surveillance in mosquitos (collected at trap locations), surveillance of WNV infection in horses, wild bird surveillance for WNV presence, and collection of human case residential addresses [20,21]. In this study, the data on disease occurrence in humans and animals and locations where WNV-positive mosquito pools are found were combined with predictor variables from geospatial data sets to develop risk maps and identify hotspots, i.e., high-risk areas of WNV.

## 2. Materials and Methods

**Figure 1:**
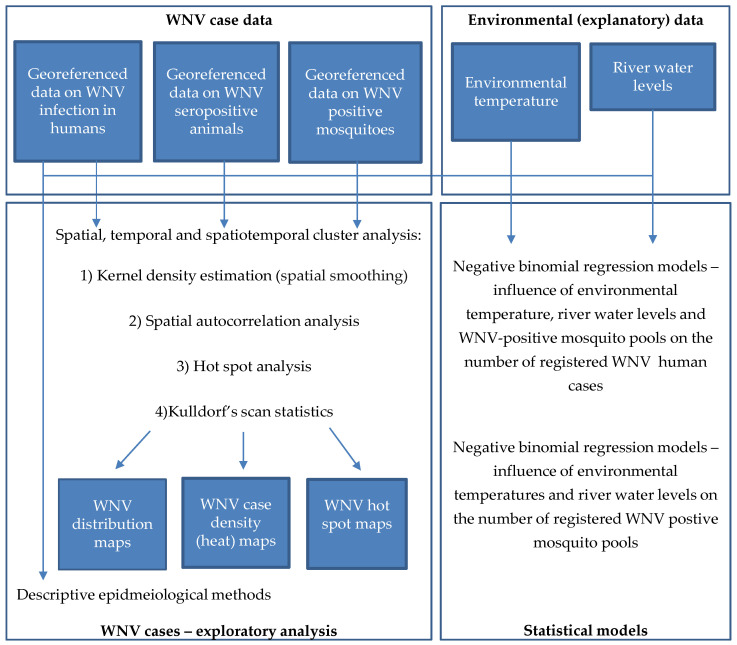
Analytical workflow of West Nile virus (WNV) epidemic explanatory analysis and risk mapping.

As shown in Figure 1, the analytical workflow involved collecting, processing, and descriptive analysis of cases of WNV infection in humans and animals; retrieving, manipulating, and processing environmental data; processing geospatial data, and; developing negative binomial regression models to measure the association between WNV infection and climatological factors. It comprises five major parts, i.e., collection and processing of epidemiological data, collection and processing of environmental data, exploratory analysis of WNV case data, statistical modeling, and mapping the distribution of WNV cases.

Cases of WNV infection and climatological factors in South Banat District, were analysed using ESRI’s ArcGIS 10.5 software (ESRI Geographic information system company, West Redlands, CA, USA), then SaTScan v.9.6 (Martin Kulldorff and Information Management Services Inc., Boston, MA, USA), IBM SPSS Statistics v.26 (IBM, SPSS Inc., New York, NY, USA), and Pavanaarekh v.5. (Envitrans Infosolutions Private Limited, Ghaziabad, India). Data were manipulated temporally for the entire period and through spatial programming. Local patterns for grouping of animals, humans, and mosquitoes that were positive over time for the presence of WNV were investigated, using three types of models, from heat maps to complex space–time models.

### 2.1. Data and Sources of Information

For the spatiotemporal analysis, we collected data for the time of the onset of the disease, incidences, and geographic locations of registered cases. Those data were aggregated based on geographic locations. The final input data had to be compliant with the software in use, i.e., ArcGIS 10.5 and SaTScan v 9.6. Data on WNV cases, animals seropositive for WNV, and WNV-positive mosquito pools were georeferenced and transformed into shapefiles that were used for spatial, temporal, and spatiotemporal analysis. A variety of data sets were compiled that contained land cover information and environmental data relevant to disease transmission and the survival of WNV in nature. As base maps, polygon shapefiles and rasters of the geography of South Banat District were used [22,23].

Land cover data for South Banat District were acquired from Copernicus Land Monitoring Service (CLMS). Their CORINE Land Cover (CLC) inventory consists of an inventory of land cover in 44 classes, uses a minimum mapping unit of 25 hectares for areal phenomena, and a minimum width of 100 m for linear phenomena. Land cover data include land use, vegetation, topography, soils, and wetlands [24].

Climatic data, i.e., daily temperature, precipitation, and the water level were acquired from the Meteorological Service of the Republic of Serbia. We calculated two-weekly mean temperature (°C), total monthly precipitation (mm), and monthly river water levels (cm) for the entire study period [25]. Then, we imported these data into regression models, and tested them against registered WNV human cases and registered WNV-positive mosquito pools. The data on registered WNV infection in animals and WNV-positive mosquito pools obtained during our research were used in the analysis, as were data taken from the Institute of Public Health Pančevo and the European Center for Disease Prevention and Control (ECDC), i.e., official data on registered WNV human cases [9,20]. In negative binomial regression models, WNV human cases and data on WNV-positive mosquito pools were aggregated monthly. Negative binomial regression analysis was used to test for associations between predictors (explanatory variables) and dependent count variables, i.e., climate factors and WNV human cases and positive mosquito pools, respectively. Given that it is the variance of the dependent variables was higher than the mean (variance = 36.68, mean = 3.24, CV = 187%), the data related to WNV human cases were first tested to determine whether they followed Poisson’s theoretical distribution. The Kolmogorov–Smirnov (KS) test was used to determine whether the data on WNV human infections originates from a population with a specific distribution, i.e., the Poisson distribution. The obtained result of the KS test (asymptotic *p*-value of 0.000) confirmed that the data do not follow the Poisson distribution. Moreover, the significant deviation of variance from the mean value of registered WNV human cases (the deviance over the degree of freedom equal to 1.008) indicated overdispersion of the data. Taking into account these observations and the results of statistical tests, the negative binomial regression model was found appropriate for the analysis. The goodness of fit determined how well the selected model fits a set of observations compared to the other model (Poisson regression model). In assessing whether a given distribution is suited to a data set, the following tests were used: Likelihood test, Bayesian information criterion and Akaike’s information criterion. The omnibus test, i.e., likelihood ratio chi-square test was used to test whether the negative binomial regression model represents a significant improvement in fit compared to the null model. The results of the omnibus test showed the significant improvement of the model based on the negative binomial distribution, i.e., likelihood ratio chi-square statistic equal to 12.48, *p*-value of 0.002. The same procedure was applied when it came to data on WNV-positive mosquito pools. The regression model was validated in IBM SPSS software by performing a cross-validation method with the same data divided into two parts, i.e., the training sample which covered 55% data and the test sample which covered 45% of the data.

### 2.2. Study Area

The study area was the South Banat Administrative District with its settlements. The area is rich in running and standing waters, has a network of irrigation channels, and incorporates the Danube and Tamis rivers, with the Danube river forming a delta right next to the city of Pančevo, as well as in several locations downstream. The administrative center of the district is the city of Pančevo, which is located about 2.5 km upstream from the mouth of the river Tamis into the Danube. The district lies in the region of Banat and has a population of 293,730 inhabitants [26,27]. The area is also a significant habitat for wild birds [28]. The South Banat Administrative District extends to Serbia’s north, in the southeastern part of the Autonomous Province of Vojvodina. It is bordered by the Danube and Tamis rivers, and on the east by the state border with Romania. South Banat covers an area of 4245 km^2^ [26].

Livestock production in South Banat is heterogeneous. Different types of domestic animals are bred and held on rural holdings and industrial farms. Domestic animals in rural areas are usually reared extensively on backyard holdings, but intensive production on industrial farms is also present [29].

### 2.3. Mosquito Pooled Samples

To collect pooled mosquito samples, modified Centers for Disease Control and Prevention (CDC) mosquito light traps were set in the afternoons at selected location sites. Criteria for site selection were accessibility, an unkempt natural environment, with lots of trees and vegetation, and the vicinity of farms and backyard holdings, wetlands, temporary standing water, water-filled containers, and shady places. The mosquitoes were brought into the Laboratory for Medical and Veterinary Entomology, Faculty of Agriculture, University of Novi Sad. The mosquitoes were kept alive on dry ice to prevent virus degradation. In the laboratory, mosquitoes were sorted, identified, and grouped on a cold table. Only the *Cx. pipiens* mosquitoes (biotypes *pipiens* and *modestus*) were separated and transported on dry ice to the Department for Virology of the Scientific Veterinary Institute Novi Sad to be tested for WNV presence. Mosquitoes were tested in pools of up to 50 individuals. When traps contained more than 300 *Cx. pipiens* mosquitoes, two pools of up to 50 individuals were tested per trap.

Sampling of mosquitoes from selected localities was performed in 2017 and 2018 in four sampling periods, one in June, two in July, and one in August. A total of 80 samples were collected and tested for WNV presence.

### 2.4. Surveillance Targeting Dead Wild Birds

Dead wild birds found in the natural environment, shot targeted wild birds, and tracheal/pharyngeal swabs of wild birds, live-trapped during the ringing and other activities of bird protection societies, particularly the resident species most susceptible to infection, were collected in the period from June to December 2017 and 2018 and tested for the presence of WNV genomic RNA.

### 2.5. Blood Samples

Blood serum samples from cattle, pigs, and chickens were collected at several locations in South Banat District. The blood of domestic animals was sampled on industrial farms and backyard holdings. Sampling was performed at equal monthly intervals from March to October in 2018 and March to October in 2019. To determine seroconversion, sera were tested for the presence of specific IgG antibodies against WNV using the ELISA test (ELISA test INGEZIM West Nile Compac, INGENASA, Madrid, Spain).

### 2.6. WNV Genome Detection by Molecular Methods

Mosquito pools (up to 50 individuals in one pool) and wild bird samples (tissues and tracheal swabs) were tested for WNV RNA presence by TaqMan-based one-step reverse transcription real-time PCR (RT-qPCR) that amplified both lineage 1 and 2 strains of the virus, as described elsewhere [21].

### 2.7. Spatiotemporal Analysis

WNV infections in humans, animals, and mosquitoes were analyzed for both space and time patterns using the following analytical approaches: kernel density analysis (Parzen windows-cores), incremental spatial autocorrelation (Moran’s *I* test), hot spot analysis (Getis-ord GI* statistics), and space–time aggregation (temporal, spatial, and spatio-temporal analysis of clusters using Kulldorff spatial scan statistics).

#### 2.7.1. Kernel Density Estimation

Kernel density estimation (KDE) is a method used to create “heat maps”, commonly applied in epidemiological research of WNF and other diseases [16,30,31,32,33]. KDE was performed using the spatial analysis/kernel density tool of the ArcGIS desktop 10.5 software package. The KDE tool was used to calculate the density of cases of WNV infection in the immediate vicinity (neighbourhood) around each entity (point object on the map that represents a registered case of WNV infection). It takes known quantities, i.e., the numbers of WNV infections, and extrapolates them across the landscape based on the quantity that is measured at each location and the spatial relationship of the locations of the measured quantities. The KDE technique creates a smoothed map of the density of cases of WNV infections at each location where positive cases were registered. The output raster surface is transformed into contour maps to allow for overlay onto other geographic layers. Although different sizes of kernels were analyzed, results for 660 m are reported, which was estimated as a standard distance by ArcGIS software itself. It is the default search radius (bandwidth) based on Silverman’s rule-of-thumb bandwidth estimation [33,34,35].

#### 2.7.2. Spatial Autocorrelation Analysis

Spatial autocorrelation analysis is a precondition for the in-depth spatial analysis of WNV infections. This method included global autocorrelation and local autocorrelation. The global autocorrelation was used to analyze whether the attributes specified in the study area were relevant at the level of the entire South Banat District, while the latter accurately determines where such attributes are gathered and reveals the spatial distribution pattern and the approximate spatial aggregation range. Moran’s index *I*, which ranges from −1 to +1, is an indicator of global autocorrelation analysis. A value close to 1 or to –1 indicates, respectively, a strong positive or negative spatial autocorrelation. Moran’s I can be tested based on Z-score and *p*-value, to determine whether or not the null hypothesis, that the incidence of WNV infection was randomly scattered in space, should be rejected [36,37].

#### 2.7.3. Hot Spot Analysis

Hot spot areas are concentrations of incidents within a limited geographical area that appear over time. Hot spot analysis is also statistically known as cluster analysis. The most intuitive type of cluster is when only the location of incidents is considered. Thus, the location with the highest number of incidents is considered to be a hot spot [38]. In our investigation, one cluster represented a group, i.e., a series of cases of WNV infection in one geographical area in different animal species and humans.

The hot spot analysis was based on a statistical calculation known as Getis-ord Gi * statistics. Getis-ord Gi * statistics are based on the calculation of *z*-statistics and *p*-values, where *z*-statistics determine whether the examined values of a particular attribute of a cluster entity are above or below the average values at the level of the whole geographical area (necessary to determine “hot” or “cold” points), while *p*-values determine whether the grouping of units into clusters is the result of a random event or a consequence of some external influence, a phenomenon that conditions the grouping. Hot spot analysis was performed using the cluster mapping/spatial statistics tools of the software package ArcGIS desktop 10.5 [39,40,41,42].

#### 2.7.4. Space–Time Aggregation

The most sophisticated space–time analyses were conducted using SaTScan v9.6., a method that has been previously used in many WNF studies to analyze spatial, temporal, and spatio-temporal data using spatial, temporal, or spatio-temporal scanning. It detects geographical areas where WNV cases are grouped more densely than their usual distribution. Within the cluster, all cases of the disease are associated with the same epidemic [15,43,44,45,46,47]. An important feature of spatial scanning is the ability to detect cluster locations and make inferences about clusters, that is, locating the geographic areas of the most likely clusters and the secondary clusters [48].

## 3. Results

### 3.1. Epidemiological Characteristics of WNV Outbreaks in 2017, 2018, and 2019

#### 3.1.1. Descriptive Statistics

**Figure 2:**
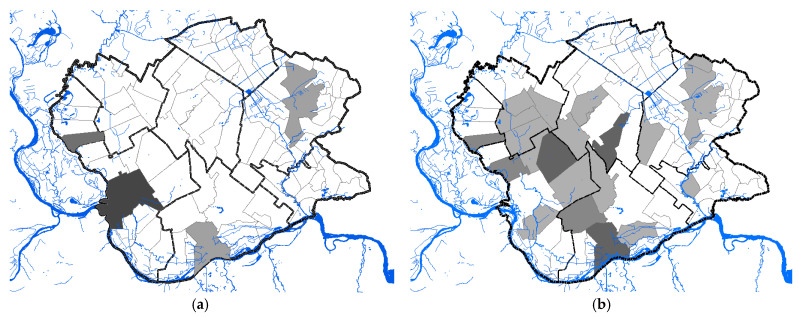

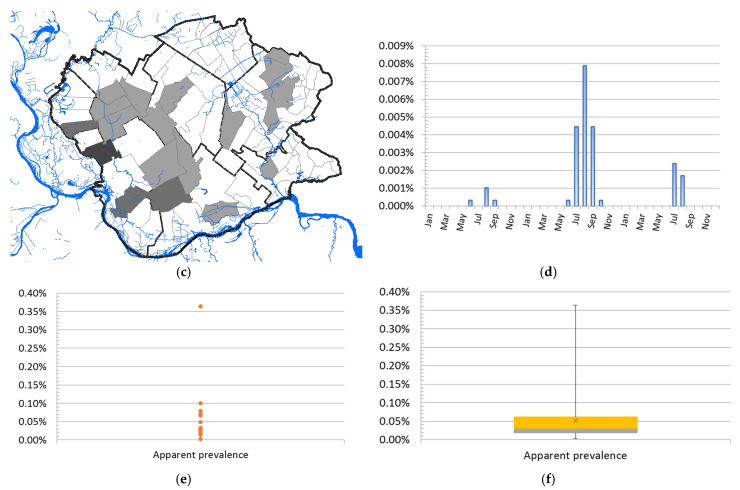
Epidemiological characteristics of West Nile virus outbreaks in 2017, 2018, and 2019: (**a**) Distribution of WNV human cases in 2017; (**b**) distribution of WNV human cases in 2018; (**c**) distribution of WNV human cases in 2019; (**d**) incidence risk of WNV infection in humans during the study period; (**e**) dot plot of WNV prevalence in 2018; (**f**) box plot of WNV prevalence in 2018.

A total of 68 human clinical, laboratory-confirmed cases of WNV infection were included in this study. The case number of WNV in humans maintained a seasonal variation in the study period, ranging from 0.24 to 2.48 cases per 100,000 person-months at risk. We estimated that the average incidence rate of WNV in 2017 was 0.24 per 100,000 person-months at risk, whereas, in 2018, the average incidence rate was 2.48 per 100,000 person-months at risk, and in 2019 it was 0.58 per 100,000 person-months at risk. The period prevalence in 2017 was 0.0017%, while in 2018 it was 0.017%, and in 2019 it was 0.0041%. The period prevalence in 2018 in populated areas ranged from 0.0028% to 0.3636% (Supplementary Figure S10). The largest number of registered WNV cases was recorded in Pančevo, a total of 18 cases. The highest prevalence of WNV infection was recorded in the villages of Mali Žarm and Dupljaja, 0.36% and 0.10%, respectively. Figure 2 shows the distribution of clinical cases of WNV infection and the epidemiological characteristics of the WNV human outbreaks in 2017, 2018, and 2019 in the South Banat District.

#### 3.1.2. Seasonality

**Figure 3:**
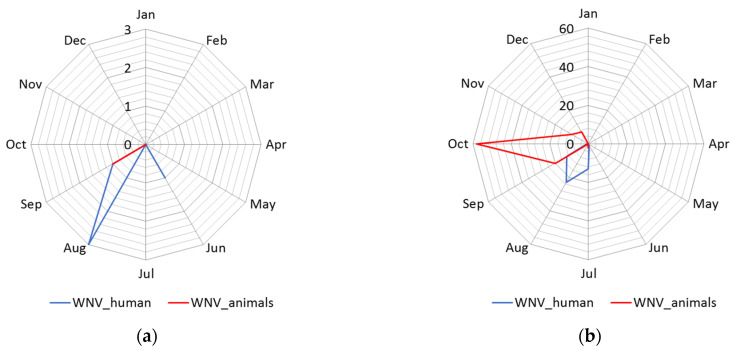

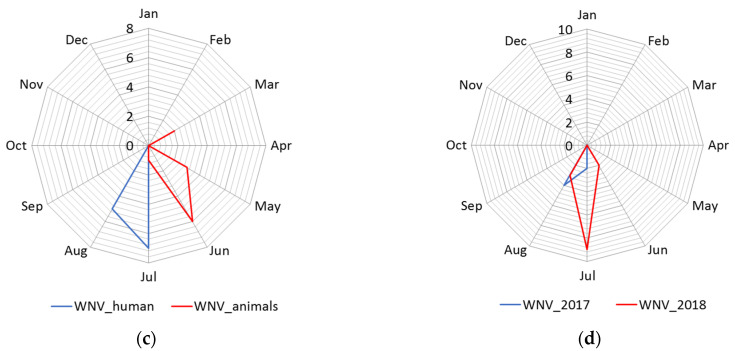
Seasonality of the West Nile virus WNV infection registered in South Banat District in different months: (**a**) Cases of WNV infection in humans and animals in 2017; (**b**) cases of WNV infection in humans and animals in 2018; (**c**) cases of WNV infection in humans and animals in 2019; (**d**) WNV infected mosquitoes in 2017 and 2018.

The distribution of human cases of WNV infection displayed a clear seasonal pattern (Figure 3). During the WNV transmission season in 2017, human cases of WNV infection were recorded between June to September, whereas in 2018 they occurred between June to October (Figure 3). The highest number of human cases were recorded in 23 August 2018 in total. In the following transmission season in 2019, although a significantly smaller number of cases of infection were registered, the appearance of the disease was also seasonal. Thus, human cases of WNV infection were recorded between July to August 2019, and the highest number of cases was recorded in July, i.e., seven in total (Figure 2 panel d).

The average ambient and average ambient maximum temperature of the two weeks before the first WNV human case was detected in 2017 were 22.5 °C and 28.26 °C, respectively, in 2018 were 20.84 °C and 25.96 °C, respectively, and in 2019 were 23.35 °C and 26.61 °C, respectively. The average ambient and average ambient maximum temperature of the two weeks before human cases terminated ranged between 23.35 °C to 26.05 °C and 26.61 °C to 32.31 °C, respectively. The highest incidence was observed when the two-week average ambient and average ambient maximum temperature were 26.73 °C and 26.73 °C, respectively. Negative binomial regression analysis was used to estimate relationships between registered WNV human cases and monthly average ambient temperatures, i.e., minimum average temperatures, average temperatures, and maximum average temperatures. Regression analysis proved a positive association between environmental temperature, the number of WNV-positive mosquito pools, and the registered number of WNV human cases. The estimated negative binomial regression coefficients for the model predictors variables, i.e., average minimum temperature, and WNV-positive mosquito pools, were positive and significant, indicating that these factors are significant predictors of infection in humans. The negative binomial coefficients for the minimum average temperature and WNV-positive mosquito pools were β_min_t⁰_ = 0.251 and β_mosq.1_ = 0.335, respectively, bound with 95% Wald confidence interval (CI) of 0.072 to 0.431 and 95% CI of 0.029 to 0.641, respectively. The estimated *p*-values were.006 and.032, respectively. The results showed that for a one-unit change in the predictor variables, the difference in the logs of expected counts of the WNV human cases is expected to change by the respective regression coefficient, provided that the other predictor variables in the model are held constant. Concerning the incidence rate ratio (IRR) the exponential value of the regression coefficient β_min_t⁰_ for minimum average temperature indicates that every increase of average minimum temperature for one degree of celsius would increase the incidence rate of WNV infection in humans by a factor of 1.286 or 28.6% and for predictor variable WNV-positive mosquito pools the exponential value of the coefficient β_mosq.1_ indicates that every increase of registered WNV-positive mosquito pools by one would increase the incidence rate of WNV infection in humans by a factor of 1.398 or 39.8%, provided that the parameter of the minimum average temperature is unchanged. The negative binomial regression coefficients for the average temperature and WNV-positive mosquito pools were β_averge_t⁰_ = 0.223 and β_mosq.2_ = 0.331, respectively, bound with 95% CI of 0.055 to 0.392 and CI of 0.028 to 0.635, respectively, while for the average maximum temperature and WNV-positive mosquito pools were β_max_t⁰_ = 0.219 and β_mosq.2_ = 0.303, respectively, bound with 95% CI of 0.043 to 0.395 and 95% CI of 0.004 to 0.602, respectively.

The results of the regression analysis show that fluctuations, ie changes in the water level of the Danube, affect the number of registered cases of WNV infection, both in humans and mosquitoes. The estimated negative binomial regression coefficients for the minimum, average, and maximum water level, were negative and significant, which practically means that as the river level decreases, the number of registered cases increases. The following values of regression coefficients concerning the variables minimum water level, average level and maximum water level were obtained: β_water_min_ = −0.021 (95% CI −0.038 to −0.003, *p*-value 0.02) β_water_average_ = −0.0019 (95% CI −0.034 to −0.005, *p*-value 0.009) and β_water_max_ = −0.013 (95% CI −0.023 to 0.003, *p =* 0.012).

Regression analysis proved a positive association between environmental temperature and the number of WNV-positive mosquito pools. The estimated negative binomial regression coefficient was positive and significant, indicating that this factor is a significant predictor. However, the precipitation was negative and insignificant, so as such it was excluded from the model. Instead of this predictor, the model includes the water level of the Danube as a predictor. The following values of the coefficients were obtained by regression analysis: β_min_t⁰_ = −0.184 (95% CI −0.005 to 0.364, *p =* 0.04) β_water_max_ = −0.013 (95% CI −0.026 to −0.001, *p =* 0.04). The estimated negative binomial regression coefficient for predictor—the water level of the Danube was negative and significant, indicating that this factor is a significant predictor.

The model was validated by the cross-validation method with satisfactory results. For the sample test that included 45% of the data, Spearman’s rank correlation coefficient, ρ was 0.708 (*p =* 0.033), while Kendall’s compliance coefficient, τ was 0.591 (*p =* 0.045). For validation purposes, the data on the number of registered WNV human cases of infection in humans, data on WNV-positive mosquito pools, and temperature were used. For the training sample with 55% of the data the correlation coefficient ρ was 0.628 (*p =* 0.029) and Kendall’s τ was equal to 0.557 (*p =* 0.018). By comparing the actual values of the WNV human cases and the values predicted by the model were calculated bias, mean average error (MAE), normalised MAE, mean square error (MSE) I root mean square error (RMSE). The following values were obtained: bias = 1.53, MAE = 2.07, NormMAE =0.64, MSE = 16.03, and RMSE = 4.08.

More information on the analysis of the effects of environmental temperatures on the risk of WNV transmission is available in the Supplementary Material (Supplementary Tables S1–S15).

#### 3.1.3. Geographical Distribution and Abundance of WNV-Positive Mosquitoes

**Figure 4:**
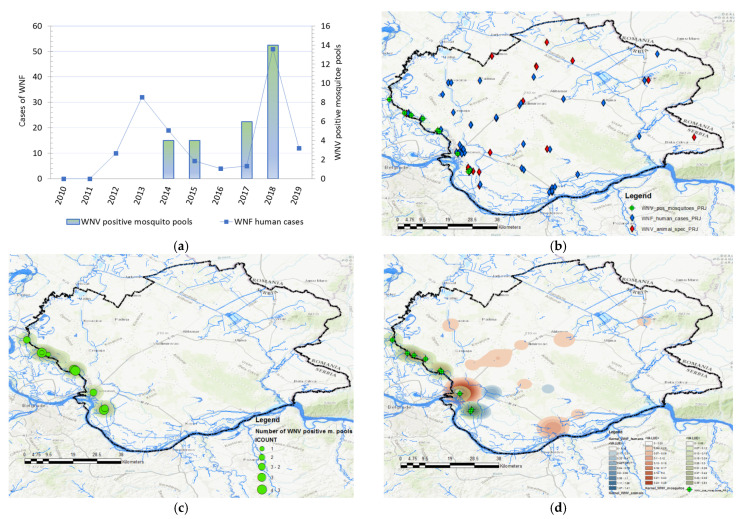
Distribution and abundance of the West Nile virus (WNV) human cases and WNV-positive mosquito pools: (**a**) Frequency distribution of human cases of WNV infection, and WNV-positive mosquito pools in 2014, 2015, 2017, and 2018; (**b**) distribution of registered cases of WNV infection in humans (blue), domestic animals (red), in 2017, 2018, and 2019 and mosquitoes (green) in 2017 and 2018; (**c**) geographical distribution and abundance of the WNV-positive mosquito pools in 2017 and 2018; (**d**) overlay of kernel density clusters of WNV in humans (red), domestic animals (pink), and clusters of WNV-positive mosquitos (blue).

Data in Figure 4 displays the distribution of WNV-positive mosquito pools and the distribution of human cases between 2010 and 2019. Mosquitoes were surveilled for the presence of WNV in 2014, 2015, 2017, and 2018. During the 2017 and 2018 surveillance seasons, significantly higher numbers of WNV-positive mosquito pools were registered, especially in 2018. In 2017, the virus was detected in mosquitoes at a total of 5 out of 10 locations, while in 2018, circulation was proven at 9 out of 10 tested locations. The frequency of WNV-positive mosquito pools was used to create kernel density maps for each year (2017 and 2018) and these were compared with kernel density maps of WNV infection in humans and animals (Figure 4 panels (e) and (f)). By comparing clusters, we noticed a positive match between the clusters of WNV-positive mosquitoes and the clusters of registered human and animal cases of infection, shown in Figure 4 panels (c) and (d). Moreover, the clusters of WNV-positive mosquito pools, which were classified by the KDE technique into the category of very high-density clusters, coincided with the clusters of WNV infection in humans and animals, which were also categorized as high-density clusters or very high-density clusters. Matches were not observed only in those mosquito sampling localities where there were no domestic animals, and that were distant from populated areas. The increased higher numbers of registered human and animal cases correspond to the areas with a higher density of WNV-positive mosquito pools.

#### 3.1.4. The Water Level of the Danube and Tamiš Rivers

**Figure 5:**
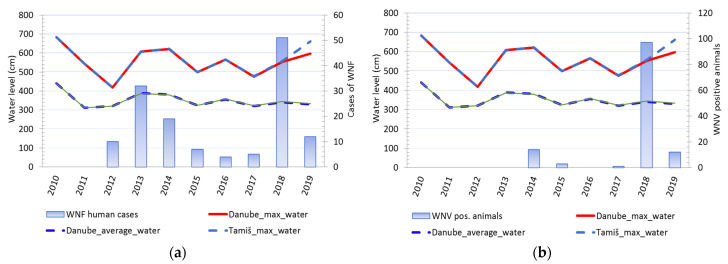
Water level fluctuations and registered cases of West Nile virus (WNV) infection in humans and animals over 10 years: (**a**) Water level fluctuations and registered number of WNV human cases; (**b**) water level fluctuations and registered cases of WNV seropositive animals.

**Figure 6:**
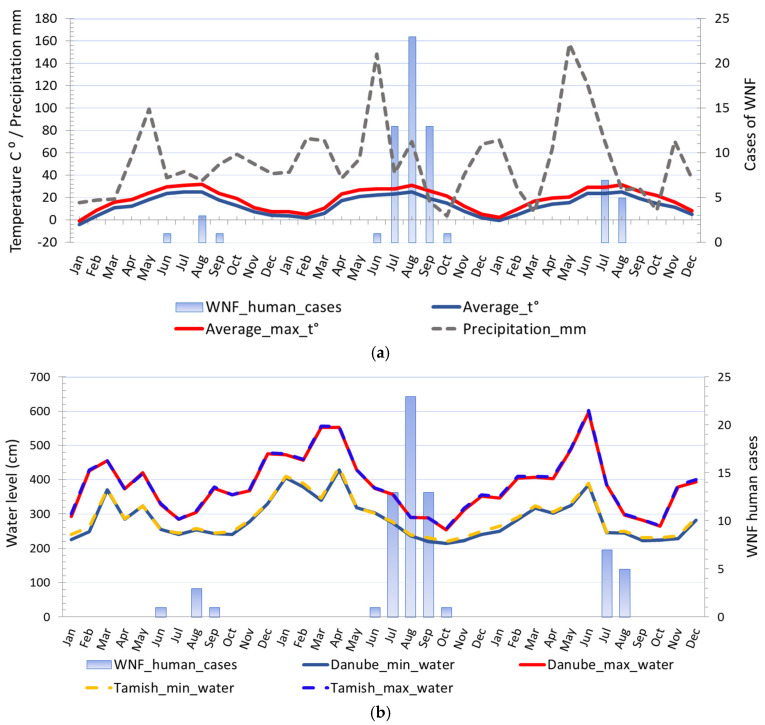
Comparison of different climatic risk factors and distribution of registered WNV human cases in 2017, 2018, and 2019: (**a**) Seasonal fluctuations in temperature, precipitation, and registered cases of WNV infection in humans; (**b**) seasonal fluctuations in water levels and registered cases of WNV infection in humans.

Comparison of changes in water levels in the Danube and Tamis rivers and cases of WNV showed that changes in water levels in rivers were accompanied by a change in the number of registered clinical cases of WNV in humans. Except in 2016, every increase in water levels and a sharp decline in river levels was accompanied by a consequent rise in the number of sick people. An identical pattern was observed for cases of infection in tested animals (Figure 5). It was notable that animal cases of infection appear after the end of the period of decline of previously high water levels. (Figure 6).

For more information on the analysis of effects of river water levels and precipitation on the risk of WNV transmission see Supplementary Material (Supplementary Tables S15–S19).

#### 3.1.5. Results of Seroconversion Tests in Domestic and Wild Animals

**Figure 7:**
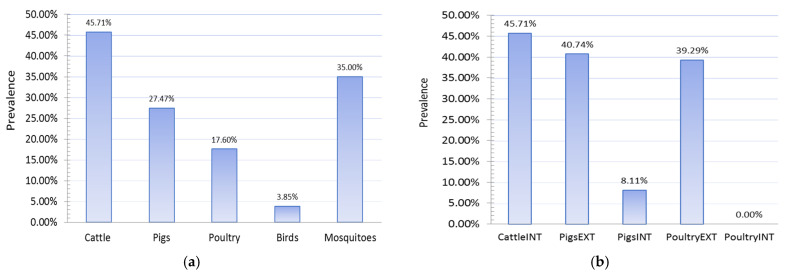
Seroprevalences showing (**a**) PCR detection of West Nile fever (WNV) in wild animals and mosquitoes and (**b**) WNV infection in domestic animals, 2018. INT—intensive farm production; EXT—extensive production on rural holdings.

The study of the seroprevalence of WNV in domestic animals showed high prevalences of infection in domestic animals, especially those raised on rural holdings in an extensive manner (Figure 7). The highest level of seroprevalence was recorded in cattle in 2018, followed by pigs raised on rural holdings, 45.71%, and 40.74%, respectively. Pigs reared on rural holdings are 7.79 times more likely to be infected with WNV than pigs on industrial farms. This odds ratio (OR 7.79, 95% CI 2.12 to 28.57) means the type of production was recognized as an exposure risk factor, and this category of animals is more prone to infection. In 2018, chickens kept on rural holdings also had a high prevalence of infection (*p* = 39.29%), while no case of infection was detected in any chicken kept on industrial farms. Chickens reared on rural holdings are more likely to be infected with WNV than chickens on industrial farms.

### 3.2. Results of Spatiotemporal Analysis

#### 3.2.1. Cluster Analysis

**Figure 8:**
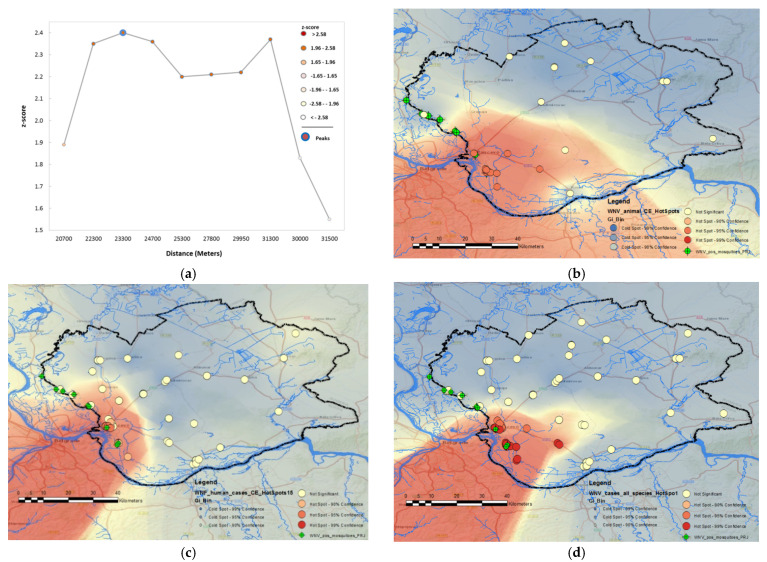
Geospatial hot spot analysis of the formation of “hot” spots of cases of WNV infection in animals and humans in 2017, 2018, and 2019: (**a**) Global spatial autocorrelation analysis of WNV outbreaks (Global Moran’s I test); (**b**) WNV hot spot analysis of cases of infection in animals; (**c**) WNV hot spot analysis of human cases; (**d**) combined WNV hot spot analysis of infection in humans and animals.

The global spatial autocorrelation analysis of the average annual incidence of WNV infection in South Banat District in 2018 suggested that a significant positive spatial autocorrelation existed. By calculating the global autocorrelation, we determined the relevance of attributes at the level of the entire study area, whereby a perimeter of 23.3 km indicates distances where spatial processes that promote clustering are most pronounced. As shown in Figure 8 panel (a), the test results indicate that a statistically significant grouping of clusters occurs in a diameter of 23.3 km (Global Moran’s I test: the first peak of *z*-score 2.40, *p*-value of 0.016 for a distance of 23.3 km). The results of the hot spot analysis and purely spatial cluster analysis showed a variation in the spatial distribution of WNV cases in South Banat County, with most high-risk clusters located nearby water streams (Figure 8). Red, orange, and light orange dots on panels (b), (c), and (d) represent clusters that are were located as “hot” spots, i.e., they form a group of clusters in which grouping is not the result of a random event but occurs as a result of environmental risk factors influence. The level of statistical significance of cluster grouping was graded in the interval of 90–99%. Yellow dots represent clusters whose grouping is the result of a random event and cases are not interconnected.

#### 3.2.2. Space–Time Aggregation

Kulldorff’s spatial cluster analysis indicated that the cases of WNV were not randomly distributed in space and time in 2017, 2018, or 2019. A total of six significant purely spatial clusters (Table 1), one purely temporal cluster (Table 2), and two significant and one secondary spatio-temporal clusters were discovered (Table 3). The most likely spatial clusters were mainly found in the southwestern part and central part of South Banat District, nearby main water streams and dense vegetation (the Danube and Tamiš rivers, and Ponjavica Nature Park), including 18 settlements. For more information on spatial cluster analysis see Supplementary Material (Supplementary Figures S4–S9).

## 4. Discussion

This research revealed the epidemiological characteristics of WNV infection in the South Banat District. We have analyzed the changes occurring concerning the spatial, temporal, and spatiotemporal trends during the study period between 2017–2019, using the GIS spatial analysis technique, scan statistics, descriptive analysis of WNV case data, and statistical modeling.

Our study confirmed that the most important spatial risk factors for WNV infection in South Banat District were the nearness of waters (water streams, standing waters, and the network of irrigation canals), and proximity to WNV-positive mosquito sites. In addition, proximity to wild bird habitats and the presence of dense vegetation cover were important risk factors. The graphical representation of the land cover of the South Banat District is shown in Supplementary Materials (Supplementary Figures S8 and S9). Thematic maps, created using the above-mentioned risk factors, identified WNV hot spot locations in all of the analyses. Areas that were identified as primary and secondary hot spots were mostly located nearby water streams, in the southwestern or central parts of the South Banat District.

We found the main risk factors of the epidemic in humans in 2018 were the high environmental temperatures and the sharp decline of previous maximum water levels in July and August, i.e., the peak of the epidemic correlated with the lowest water levels of the Danube and Tamis rivers (monthly average minimum and monthly average maximum temperature: 17.8 °C and 31.7 °C respectively, and river water levels of the Danube and Tamis rivers of 236 mm and 238 mm, respectively) [49].

However, although a similar pattern of the interrelationship of these risk factors with the peak of the epidemic was observed in 2017 and 2019, the number of registered human cases was significantly lower than in 2018. At the peak of the epidemics in August 2017 and 2019, only three and five cases of the disease were registered, while in 2018, 23 cases were registered. In terms of hydrology, 2017 and 2019 differed from 2018 in that in 2017 and 2019, no extended periods of maximum water levels were registered, preceding dry summer periods with minimum water levels; this was not the case in 2018.

The results of negative binomial regression analysis show that temperature is a significant risk factor, i.e., a predictor of the appearance of mosquitoes infected with WNV. At the time of the onset of the disease in 2018, the average ambient and average ambient maximum temperature ranged between 20.84 °C to 23.35 °C and 25.96 °C to 28.26 °C, respectively. The highest incidence was observed when the two-week average ambient and average ambient maximum temperature were 26.73 °C and 26.73 °C, respectively. It is important to note that 2018 and 2019 were the warmest years in the history of meteorological measurements in Serbia. The prolongation of the warmest summer period and the warmer autumn period coincides with the early spring migration of birds, thus extending the time duration for infection of mosquitoes with WNV, which increases the chances of transmitting the disease to humans and animals [50]. High environmental temperature increases the risk of WNV transmission [51,52]. High environmental temperature promotes higher growth rates of mosquitoes and makes the extrinsic incubation period shorter; the gonotrophic cycle (the time required to produce eggs after a blood meal) is also shorter [53,54]. Based on negative binomial regression analysis, we found that variations in the number of registered WNV human cases can be partially explained and related to temperature variations and the increased number o WNV-positive mosquito pools. These two factors have proven to be good predictors of the growth in the number of infected people. On the other hand, the declining level of the Danube also shows a positive association with the increase in the number of patients and the number of registered WNV-positive mosquito pools.

For more information on water levels of the Danube and Tamis rivers, and temperature regimes see Supplementary Materials (Supplementary Tables S1–S4 and Figures S1 and S2).

Besides climate factors and analysis of seasonality, in this study, the risk factors were also evaluated at spatial scales, based on the primal assumption that the flight range of mosquitoes is crucial for transmission and mosquitoes are the main transmission force. The landscape of the South Banat District, such as vegetation, soils, wetlands, and topography, provides favorable conditions for both mosquito and wild bird multiplication. The high-risk zones include plenty of agricultural areas, farms, rural holdings, wetlands [55], and urban areas with inland marshes and small forest zones [52].

An important element for performing either risk analysis or hot spot analysis is determining the radius of the grouping, i.e., the boundaries (perimeter) of the geographical area on which the grouping of entities into clusters is analyzed and which determines their significance. The hot spot analysis for the numbers of cases indicated that the disease is most prevalent in locations nearby water streams. By comparing the grouping of clusters, we noted a positive match between the clusters of WNV-positive mosquitoes and the clusters of registered human and animal cases of infection. The clusters of WNV-positive mosquitoes, which were classified by the KDE technique into the category of very high-density clusters, coincided with the clusters of WNV infection in humans and animals, which were also categorized as very high-density clusters or high-density clusters.

Possible factors that might influence such a wide grouping (grouping radius) of statistically significant clusters of WNV occurrence in South Banat District are the intensity of prevailing winds and the abundance of running and standing waters. Winds have a great effect on mosquito dispersion. Kay and Farrow reported flight distances of 648 km for *C. annulirostris* as a result of wind blow [56]. Other authors have reported the results of similar research where substantial distances were identified, i.e., 200 km for *Culex tritaeniorhynchus* [57], 280 km for *Anopheles pharoensis* [58], 500 km for *C. tritae-niorhynchus* [59], 740 km for *A. vexans* [60], and even 850 km for *Cx. pipiens pipiens* [61]. Verdonschota and Besse-Lototskaya reported mean flight distances of 10.97 km for genus Culex [62].

South Banat District is flat and characterized by a high frequency of winds. The highest frequency of wind occurrence is the southeast wind (košava) which occurs at 306 ‰, followed by the northwest wind with 255‰, while the lowest frequency of occurrence is the north wind 48‰ and the northeast 44‰. The prevailing southeast wind most often occurs in autumn 368‰, and least often in summer 196‰. The highest frequency of silences (Calme) is 143‰ in May and the lowest in November 51‰. As for the wind speed, the highest average annual speed for the area has the wind that blows from the east-southeast direction 3.3 m/s, and the lowest south-southwest with a speed of 1.7 m/s. The graphical representation of the dominant winds is shown in Supplementary Materials (Supplementary Figure S3 and Table S20).

In the study area, a total of six significant purely spatial clusters, one purely temporal cluster, and two significant spatio-temporal and one secondary spatio-temporal clusters were discovered. In most situations, except in 2017, the human cases were preceded by the detection of WNV in animals and mosquitoes. In 2017, the detection of WNV-positive mosquitoes preceded the registration of the first cases of infection in humans by 55 days, while in 2018, this period was 9 days. In sentinel animals in 2017, contact with the virus was detected three months and 3 days after the registration of the first human case. However, in 2018, contact with the virus was detected 9 days before human infection, and in 2019, a full two months earlier.

To better understand the history of WNV epidemics in the study area, we additionally used the data provided by ECDC [9] and analyzed the number of infected people in the designated geographical area in the period between 2012–2019. It was notable that after the sudden appearance of the disease in 2012 and the further increase of the epidemic in 2013, there was a significant decrease in the registered cases in the following year, as was the case in 2019. The numbers of human cases per year were as follows: *n* = 10 in 2012, *n* = 32 in 2013, *n* = 19 in 2014, *n* = 7 in 2015, *n* = 4 in 2016, *n* = 5 in 2017, *n* = 51 in 2018 and *n* = 12 in 2019. The mean of the WNV case numbers was 17.5 cases per year in the period. The differences of the cases from the mean were: −7.5 in 2012, 14.5 in 2013, 1.5 in 2014, −10.5 in 2015, −13.5 in 2016, −12.5 in 2017, 33.5 in 2018, and −5.5 in 2019. This decline in human cases could be partly explained if many people had already been in contact with the virus and earned immunity, which made them less susceptible to the virus [21] but this assumption needs additional research. Variations that have emerged over the last decade might also be explained if birds re-introduce WNV into South Banat District from year to year.

Our research indicated that in creating a surveillance model for WNF, the primary concern is to consider the behavior and the requirements of every element in the disease transmission chain. Climate factors might be the main determinant of WNV distribution and predictors of endemicity, but in other situations, climate factors are not sufficient to explain the observed distribution of WNV cases. Precipitation and water levels of main rivers have an important influence on the mosquito abundance on main rivers, local water streams, and especially standing waters. Water abundance also greatly affects the habitats of wild birds, which are important for maintaining the virus in nature. The epidemic process is conditioned by the action of several necessary factors in the disease. Winds were identified as a likely risk factor, taking into account wind effect on vector dispersion. However, each of these factors is not enough to lead to the disease on its own, so each risk assessment and prediction is conditioned by observing and researching all these risk factors together. Taking into account the substantial presence of domestic animals raised in extensive production mode, our study leads us to the conclusion that the WNV surveillance system, besides horses and chickens, should be augmented by surveilling the virus in domestic animals kept on rural holdings, particularly cattle, pigs, and chickens. Since these animals live in conditions of very low levels of biosecurity measures, they are significantly more exposed to the vectors and WNV than domestic animals on industrial farms. Animals on rural holdings are relatively easy to trace and sample and do not have high surveillance costs. WNV, despite the decline in the number of human cases in 2019, remains a threat to the human population in the South Banat District. For the disease to be successfully monitored and detected in time, it is necessary to conduct constant monitoring of the presence of the virus in natural reservoirs and sentinel species, monitor and analyze climatological risk factors and be especially focused on indicators of the presence of the virus in nature.

## 5. Conclusions

We found the main risk factors of the epidemic in 2018 were the high environmental temperatures and the sharp decline of previous maximum water levels in July and August. The temperature is a significant risk factor and predictor of WNV infection in humans and mosquitoes. We found that variations in the number of registered WNV human cases can be partially explained by temperature variations and associated with the increased number of WNV-positive mosquito pools, which proved to be very significant in this study and leads us to the conclusion that it is important, as part of the surveillance program in the South Banat District, to place special emphasis on monitoring the presence of WNV in mosquitoes. It is important to emphasize that almost all cases of infection in humans were preceded by the detection of WNV-positive mosquitoes. Using statistical models, such as regression models, it is possible to quantify risk factors, especially climatic factors, and identify those that are not significant as indicators to be monitored. The WNV high-risk zones include plenty of agricultural areas, farms, rural holdings, wetlands, and urban areas with inland marshes and small forest zones. The hot spot analysis for the numbers of cases indicated that the disease is most prevalent in locations nearby water streams. By comparing the grouping of clusters, we noted a positive match between the clusters of WNV-positive mosquitoes and the clusters of registered human and animal cases of infection. In most situations, the human cases were preceded by the detection of WNV in animals and mosquitoes. The epidemic process is conditioned by the action of several necessary factors in the disease. Winds were identified as a likely risk factor, taking into account wind effect on vector dispersion. However, each of these factors is not enough to lead to the disease on its own, so each risk assessment and prediction is conditioned by observing and researching all these risk factors together. Taking into account the substantial presence of domestic animals raised in an extensive production way, our study leads us to the conclusion that the WNV surveillance system, besides horses and chickens, should be augmented by surveilling the virus in domestic animals kept on rural holdings, particularly cattle, pigs, and chickens. For the disease to be detected in time, it is necessary to conduct constant monitoring of the presence of the virus in natural reservoirs and sentinel species. Climatic risk factors should be constantly monitored and analyzed.

## Figures and Tables

**Table 1:** SatScan^TM^ purely spatial cluster analysis. The clusters of WNV cases detected by using purely spatial cluster statistics.

Cluster	* Total Locations	Radius, km	Population	# WNV Cases	Expected Cases	O/E	RR	LLR	*p*-Value
1	5	9.16	788	84	4.26	19.74	37.63	193.78	<1 × 10^−17^
2	18	27.90	1907	39	10.30	3.78	4.60	25.92	2.1 × 10^−10^
3	5	12.67	29	7	0.16	44.28	46.12	19.83	5.7 × 10^−8^
4	4	12.82	777	17	4.20	4.05	4.38	11.47	0.00012
5	8	13.23	936	16	5.05	3.17	3.39	7.86	0.0035
6	3	15.93	38	3	0.20	14.73	14.97	5.30	0.037

O/E—observed expected ratio; RR—relative risk; LLR—log-likelihood ratio; #-Number of West Nile virus cases. * For territorial affiliation see Supplementary Material (Supplementary Figure S4).

**Table 2:** SatScan^TM^ purely temporal cluster analysis. The clusters of WNV cases detected by using purely temporal cluster statistics.

Cluster	* Total Locations	# WNV Cases	Expected Cases	O/E	RR	LLR	*p*-Value
1	all	76	2.78	27.35	48.21	197.04	0.001

O/E—observed expected ratio; RR—relative risk; LLR—log-likelihood ratio; #-Number of West Nile virus cases. * For territorial affiliation see Supplementary Material (Supplementary Figures S4 and S6).

**Table 3:** SatScan^TM^ spatio-temporal cluster analysis. The clusters of WNV cases detected by using spatio-temporal cluster statistics.

Cluster	* Total Locations	Radius, km	Start Date	End Date	Population	# WNV Cases	Expected Cases	O/E	RR	LLR	*p*-Value
1	6	12.70	24 September 2018	15 October 2018	1023	48	0.07	677.35	939.16	272.35	1 × 10^−17^
2	3	5.11	5 September 2018	20 December 2018	680	39	2.85	13.70	17.42	70.09	1 × 10^−17^
3	6	15.59	17 August 2018	25 September 2018	1207	6	1.12	5.35	5.51	5.26	0.65

O/E—observed expected ratio; RR—relative risk; LLR—log-likelihood ratio; #-Number of West Nile virus cases * For territorial affiliation see Supplementary Material (Supplementary Figure S6).

## Data Availability

Publicly available datasets were analyzed in this study. Data is contained within the article or Supplementary Material. This data can be found here: [https://www.ecdc.europa.eu/en/west-nile-fever/surveillance-and-disease-data/historical], [https://www.popispoljoprivrede.stat.rs], [http://www.hidmet.gov.rs/eng/hidrologija/index.php], [https://www.zjzpa.org.rs/nadzor-nad-groznicom-zapadnog-nila], [https://land.copernicus.eu/pan-european/corine-land-cover], [https://juznobanatski.okrug.gov.rs], [https://data.stat.gov.rs/?caller=SDDB].

## Supplementary Materials

The following are available online at https://www.mdpi.com/article/10.3390/ani11102951/s1, Figure S1: Monthly temperatures in 2017, 2018, and 2019 in South Banat District, Figure S2: Precipitation and river water levels on the territory of South Banat District, Figure S3: The wind rose of prevailing winds in South Banat District, Figure S4: Spatial clusters detected by spatial scan statistic, Figure S5: Temporal cluster detected by space-time scan statistic, Figure S6: Spatiotemporal clusters detected by space-time scan statistic, Figure S7: Spatiotemporal cluster detected by space-time scan statistic, Figure S8: Distribution of registered cases of WNV infection in humans (blue), domestic animals (red), in 2017, 2018, and 2019 and mosquitoes (green) in 2017 and 2018 (distribution of cases shown on the thematic map of land cover), Figure S9: Distribution of hot spots of WNV cases in humans (blue) and domestic animals, Figure S10: Appirent prevalence of WNV human cases in stady period. (a) Box plot of WNV prevalence in 2017, (b) Dot plot of WNV prevalence in 2017, (c) Box plot of WNV prevalence in 2018, (b) Dot plot of WNV prevalence in 2018, (e) Box plot of WNV prevalence in 2017, (f) Dot plot of WNV prevalence in 2017 (red), in 2017, 2018, and 2019 in 2017and 2018 (distribution of cases shown on the thematic map of land cover); Table S1: Maximum recorded temperatures in South Banat district (2013–2020), Table S2: Maximum average recorded temperatures in South Banat district (2013–2020); Table S3: Average recorded temperatures in South Banat district (2013–2020); Table S4: Minimum recorded temperatures in South Banat district (2013–2020), Table S5: The number of registered WNV human cases and the movement of average monthly temperatures in the examined period between 2017 and 2019, Table S6: The goodness of fit statistics of the negative binomial regression model (Dependent Variable: WNV human cases)a, Table S7: Lagrange multiplier test of the negative binomial regression model (Dependent Variable: WNV human cases), Table S8: Omnibus test of the negative binomial regression model (test for overall data improvement with the negative binomial model (Compares the fitted model against the intercept-only model. Dependent Variable: WNV human cases), Table S9: Results of a negative binomial regression analysis of the influence of average minimum environmental temperature fluctuations on the number of registered cases of WNV in humans, Table S10: Results of a negative binomial regression analysis of the influence of average environmental temperature fluctuations on the number of registered cases of WNV in humans, Table S11: Results of a negative binomial regression analysis of the influence of average maximum environmental temperature fluctuations on the number of registered cases of WNV in humans, Table S12: The goodness of fit statistics of the negative binomial regression model (Dependent Variable: WNV-positive mosquito pools)a, Table S13: Omnibus test of the negative binomial regression model (test for overall data improvement with the negative binomial model (Compares the fitted model against the intercept-only model. Dependent Variable: WNV-positive mosquito pools), Table S14: Results of a negative binomial regression analysis of the influence of environmental temperature fluctuations and precipitation on the number of registered WNV positive mosquito pools, Table S15: The amount of precipitation in the period from 2013 to 2020, Table S16: Results of a negative binomial regression analysis of the influence of the minimum the Danube river water level fluctuations on the number of registered cases of WNV in humans, Table S17: Results of a negative binomial regression analysis of the influence of the Danube river average water level fluctuations on the number of registered cases of WNV in humans, Table S18: Results of a negative binomial regression analysis of the influence of the Danube river maximum water level fluctuations on the number of registered cases of WNV in humans, Table S19: Results of a negative binomial regression analysis of the influence of the Danube river maximum water level fluctuations and average minimum environmental temperatures on the number of registered WNV positive mosquito positive pools, Table S20: Distribution and length of the period of blowing of the prevailing winds in hours of blowing. Climatology, land cover of South Banat district, and statistical analyses on the effects of air temperature fluctuations and river water levels on the number of registered cases of WNV in humans and mosquitoes.
