# Supplementary material for: Spatiotemporal Analysis of West Nile Virus Epidemic in South Banat District, Serbia, 2017–2019
Source: Animals (Basel). 2021 Oct 13;11(10):2951. doi: 10.3390/ani11102951 (PMC8533022; doi:10.3390/ani11102951)
Supplement: Supplementary file 1 [file animals-11-02951-s001.zip › animals-1391347-supplementary.pdf]

Supplementary material:

Climatology, land cover of South Banat district, and statistical analyses on the effects of air temperature fluctuations and river water levels on the number of registered cases of WNV in humans and mosquitoes

1. Climatology

1.1. Temperature regime

Temperature regime as a measure of thermal conditions in South Banat District is primarily conditioned by solar radiation, geographical position, and relief. The territory of South Banat District belongs to the temperate climate. The average annual air temperature is 10.9 ^{0}C. Autumn is warmer than spring. The coldest month is January with an average monthly temperature of about 0 ^{0}C.

The warmest month is July with an average monthly temperature in the range of 20 to 22 ^{0}C. The lowest temperatures in the period 1961-1990 were registered in January (-21.0 ^{0}C). The absolute maximum temperatures in the observed period were measured in July and range from 37.1 to 42.3 ^{0}C.

Table S1. Maximum recorded temperatures in South Banat district (2013-2020).

Date	Winter	Spring	Summer	Autumn	Year
Dec 2020 / Nov 2021	20.1	18.6	---	---	20.1
Dec 2019 / Nov 2020	20.3	29.7	35.4	31.8	35.4
Dec 2018 / Nov 2019	18.5	30.2	36.5	33.8	36.5
Dec 2017 / Nov 2018	17.1	30.6	35.2	33.7	35.2
Dec 2016 / Nov 2017	22.4	30.7	39.1	33.8	39.1
Dec 2015 / Nov 2016	21	30.6	35.7	31.1	35.7
Dec 2014 / Nov 2015	15.9	32.4	37.7	36.1	37.7
Dec 2013 / Nov 2014	22.9	29.8	35.1	29	35.1
Maximum	22.9	32.4	39.1	36.1	39.1

Table S2. Maximum average recorded temperatures in South Banat district (2013-2020).

Date	Winter	Spring	Summer	Autumn	Year
Dec 2020 / Nov 2021	7.8	10.5	---	---	8.3
Dec 2019 / Nov 2020	7.7	18.80	28.3	18.2	18.7
Dec 2018 / Nov 2019	5.3	18.9	29.8	21.1	18.8
Dec 2017 / Nov 2018	6.7	20.1	28.9	19.9	18.9
Dec 2016 / Nov 2017	3.5	19.4	30.7	17.9	18
Dec 2015 / Nov 2016					

	7.3	19	28.2	17.2	18
Dec 2014 / Nov 2015	↓ 6.5	↓ 18.3	↑ 30.5	↓ 18.1	↑ 18.4
Dec 2013 / Nov 2014	↑ 9.7	↑ 19.4	↓ 27.3	↓ 17.9	↑ 20
Average maximum	6.6	18.8	29.1	18.6	18.2

Table S3. Average recorded temperatures in South Banat district (2013-2020).

Date	Winter	Spring	Summer	Autumn	Year
Dec 2020 / Nov 2021	↑ 4.3	↓ 5.3	---	---	↓ 4.5
Dec 2019 / Nov 2020	↑ 3.7	↓ 12.9	↓ 22.6	↑ 13.4	↓ 13.6
Dec 2018 / Nov 2019	↓ 2	↑ 13.3	↑ 24.1	↑ 15.1	↓ 13.7
Dec 2017 / Nov 2018	↑ 3.2	↑ 14.7	↑ 23.4	↑ 14.2	↑ 13.9
Dec 2016 / Nov 2017	↓ -0.3	↑ 13.7	↑ 24.5	↓ 12.5	↓ 12.7
Dec 2015 / Nov 2016	↑ 3.8	↑ 13.5	↓ 22.8	↓ 12	↓ 13.1
Dec 2014 / Nov 2015	↑ 2.9	↑ 13.2	↑ 24.3	↓ 13	↓ 13.4
Dec 2013 / Nov 2014	↑ 4	↓ 12.8	↓ 21	↓ 13.2	↑ 14.2
Average	2.8	13.2	23.2	13.3	13.1

Table S4. Minimum recorded temperatures in South Banat district (2013-2020).

Date	Winter	Spring	Summer	Autumn	Year
Dec 2020 / Nov 2021	↓ -11.2	↓ -5.7	---	---	-11.2
Dec 2019 / Nov 2020	↑ -7.3	↑ -2.7	↓ 6.2	↓ -2.3	↑ -7.3
Dec 2018 / Nov 2019	↓ -17.1	↑ -3	↑ 12.4	↑ 0.7	↓ -17.1
Dec 2017 / Nov 2018	↑ -8	↓ -12.8	↑ 10.9	↓ -6.2	↑ -12.8
Dec 2016 / Nov 2017	↓ -18.7	↓ -0.8	↓ 10.5	↑ -2.7	↓ -18.7
Dec 2015 / Nov 2016	↑ -12.8	↑ -0.5	↓ 11.2	↓ -3.3	↑ -12.8
Dec 2014 / Nov 2015	↓ -17.9	↓ -2.7	↑ 11.7	↑ -2.1	↓ -17.9
Dec 2013 / Nov 2014	↑ -7.8	↑ -0.8	↑ 9.9	↑ -3.1	↑ -7.8
Minimum	-18.7	-12.8	6.2	-6.2	-18.7

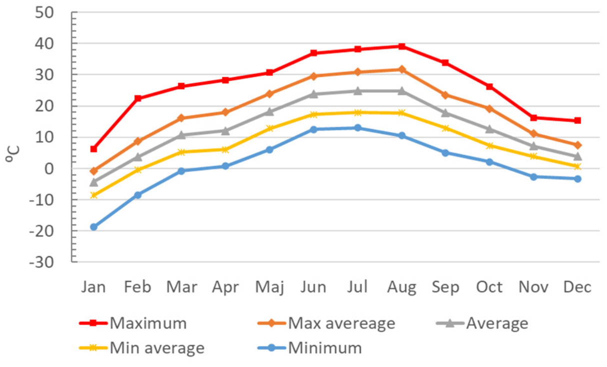

(a) 2017

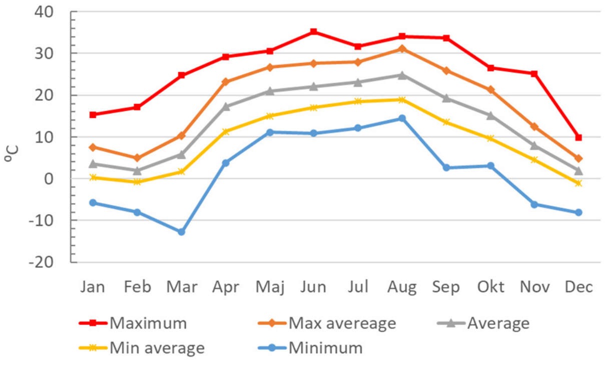

(b) 2018

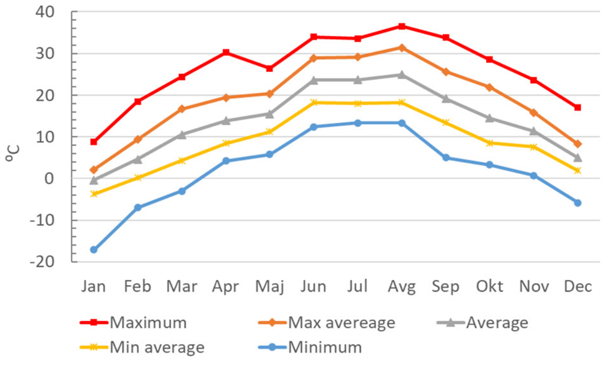

(c) 2019

Figure S1. Monthly temperatures in 2017, 2018, and 2019 in South Banat District.

1.2. Modeling the effects of temperature on the frequency of WNV infection in the period 2017-2019

Negative binomial regression analysis was used for the estimation of relationships between WNV registered human cases and registered monthly average ambient temperatures, i.e., minimum average temperatures, average temperatures, and maximum average temperatures. Results of regression modeling for the period between 2017 and 2019 revealed that variations in the number of registered WNV human cases can be explained by temperature variations and associated with the increased number o WNV-positive mosquito pools.

The results of negative binomial regression analysis show that temperature is a significant risk factor, i.e., a predictor of the appearance of mosquitoes infected with WNV and infection in humans. For measuring the association and impact of temperature fluctuations on the incidence of WNV in humans and mosquito surveillance collection pools, a negative binomial regression method was used. The results of these analyses were given in tables 5-19.

Table S5. The number of registered WNV human cases and the movement of average monthly temperatures in the examined period between 2017 and 2019.

Year	Month	WNV human cases	Average min t°	Average t°	Average max t°
2017	May	0	12.8	18.2	23.9
	Jun	1	17.3	23.8	29.5
	Jul	0	17.9	24.8	30.9
	Aug	3	17.8	24.8	31.7
	Sep	1	12.9	17.8	23.5
	Oct	0	7.3	12.6	19.1

	Nov	0	3.9	7.2	11.1
2018	May	0	15	21	26.7
	Jun	1	17	22.1	27.6
	Jul	13	18.5	23.1	27.9
	Aug	23	18.9	24.8	31.1
	Sep	13	13.5	19.3	25.9
	Oct	1	9.6	15.2	21.3
	Nov	0	4.5	7.9	12.4
2019	May	0	11.2	15.5	20.3
	Jun	0	18.2	23.6	28.9
	Jul	7	18	23.7	29.1
	Aug	5	18.2	24.9	31.4
	Sep	0	13.4	19.2	25.6
	Oct	0	8.5	14.5	21.9
	Nov	0	7.6	11.4	15.8

Table S6. The goodness of fit statistics of the negative binomial regression model (Dependent Variable: WNV human cases)[a.]

GLM model	Negative binomial regression model	Poisson regression model
Test	Value	Value
Deviance	22.445	69.334
Scaled Deviance	22.445	69.334
Pearson Chi-Square	26.343	88.712
Scaled Pearson Chi-Square	26.343	88.712
Log Likelihood[b]	-36.096	-50.713
Akaike's Information Criterion (AIC)	78.193	107.426
Finite Sample Corrected AIC (AICC)	79.604	108.838
Bayesian Information Criterion (BIC)	81.326	110.560
Consistent AIC (CAIC)	84.326	113.560

Dependent Variable: WNV human cases [a]Information criteria are in the smaller-is-better form [b]The full log-likelihood function is displayed and used in computing information criteria.

A model with lower values of AIC and BIC was chosen, i.e. negative binomial regression model.

Table S7. Lagrange multiplier test of the negative binomial regression model (Dependent Variable: WNV human cases).

	Chi-Square	df	Sig.
Ancillary Parameter[a]	0.736	1	0.391

[a]Tests the null hypothesis that the negative binomial distribution ancillary parameter equals 1.

Table S8. Omnibus test of the negative binomial regression model (test for overall data improvement with the negative binomial model (Compares the fitted model against the intercept-only model. Dependent Variable: WNV human cases).

Likelihood Ratio Chi-Square	df	Sig.
25.062	2	0.000

Table S9. Results of a negative binomial regression analysis of the influence of average minimum environmental temperature fluctuations on the number of registered cases of WNV in humans.

Parameter	B	Std. Error	95% Wald Confidence Interval	Hypothesis Test	Exp(B)	95% Wald Confidence Interval for Exp(B)

			Lower	Upper	Wald Chi-Square	df	Sig.		Lower	Upper
**(Intercept)**	**-3.597**	**1.4696**	-6.477	-0.717	5.991	1	0.014	0.027	0.002	0.488
Average_min_t	0.251	0.0916	0.072	0.431	7.530	1	0.006	1.286	1.074	1.539
WNV_mosquitoe	0.335	0.1560	0.029	0.641	4.618	1	0.032	1.398	1.030	1.898
(Scale)	1[a]									
(Negative binomial)	1[a]									

Dependent Variable: WNV_human_cases Model: (Intercept), Average_min_t^0, WNV_mosquitoe [a]Fixed at the displayed value.

Table S10. Results of a negative binomial regression analysis of the influence of average environmental temperature fluctuations on the number of registered cases of WNV in humans.

**Parameter**	**B**	**Std. Error**	95% Wald Confidence Interval		Hypothesis Test			**Exp(B)**	95% Wald Confidence Interval for Exp(B)	
			Lower	Upper	Wald Chi-Square	df	Sig.		Lower	Upper
**(Intercept)**	**-4.402**	**1.8470**	-8.022	-0.782	5.679	1	0.017	0.012	0.000	0.458
Average_min_t	0.223	0.0860	0.055	0.392	6.748	1	0.009	1.250	1.056	1.480
WNV_mosquitoe	0.331	0.1547	0.028	0.635	4.593	1	0.032	1.393	1.029	1.886
(Scale)	1[a]									
(Negative binomial)	1[a]									

Dependent Variable: WNV_human_cases Model: (Intercept), Average_t^0, WNV_mosquitoe [a]Fixed at the displayed value.

Table S11. Results of a negative binomial regression analysis of the influence of average maximum environmental temperature fluctuations on the number of registered cases of WNV in humans.

Parameter	B	Std. Error	95% Wald Confidence Interval		Hypothesis Test			Exp(B)	95% Wald Confidence Interval for Exp(B)	
			Lower	Upper	Wald Chi-Square	df	Sig.		Lower	Upper
(Intercept)	-5.539	2.4175	-10.278	-0.801	5.250	1	0.022	0.004	3.439E-05	0.449
Average_min_t	0.219	0.0899	0.043	0.395	5.956	1	0.015	1.245	1.044	1.485
WNV_mosquitoe	0.303	0.1526	0.004	0.602	3.953	1	0.047	1.354	1.004	1.826
(Scale)	1[a]									
(Negative binomial)	1[a]									

Dependent Variable: WNV_human_cases Model: (Intercept), Average_maximum_t^0, WNV_mosquitoe a. Fixed at the displayed value.

Table S12. The goodness of fit statistics of the negative binomial regression model (Dependent Variable: WNV-positive mosquito pools)[a].

**GLM model**	**Negative binomial regression model**	**Poisson regression model**
Test	Value	Value
Deviance	22.382	50.982
Scaled Deviance	22.382	50.982
Pearson Chi-Square	24.387	59.484
Scaled Pearson Chi-Square	24.387	59.484
Log Likelihood[b]	-27.625	-36.307

Akaike's Information Criterion (AIC)	61.250	78.615
Finite Sample Corrected AIC (AICC)	62.662	80.027
Bayesian Information Criterion (BIC)	64.384	81.748
Consistent AIC (CAIC)	67.384	84.748

Dependent Variable: WNV-positive_mosquitoe pools [a]Information criteria are in the smaller-is-better form [b]The full log-likelihood function is displayed and used in computing information criteria.

A model with lower values of AIC and BIC was chosen, i.e. negative binomial regression model.

Table S13. Omnibus test of the negative binomial regression model (test for overall data improvement with the negative binomial model (Compares the fitted model against the intercept-only model. Dependent Variable: WNV-positive mosquito pools).

Likelihood Ratio Chi-Square	df	Sig.
6.252	2	0.044

Table S14. Results of a negative binomial regression analysis of the influence of environmental temperature fluctuations and precipitation on the number of registered WNV positive mosquito pools.

Parameter	B	Std. Error	95% Wald Confidence Interval		Hypothesis Test			Exp(B)	95% Wald Confidence Interval for Exp(B)	
			Lower	Upper	Wald Chi-Square	df	Sig.		Lower	Upper
(Intercept)	-2.719	1.9830	-6.605	1.168	1.880	1	0.170	0.066	0.001	3.214
Average_min_t	0.293	0.1444	0.010	0.576	4.111	1	0.043	1.340	1.010	1.779
Precipitation_mm	-0.029	0.0186	-0.065	0.007	2.467	1	0.116	0.971	0.937	1.007
(Scale)	1[a]									
(Negative binomial)	2.353	1.4005	0.733	7.555						

Dependent Variable: WNV-positive mosquito pools Model: (Intercept), Average_min_t, Precipitation_mm [a]Fixed at the displayed value.

1.3. Modeling the effects of river water levels on the frequency of WNV infection in the period 2017-2019.

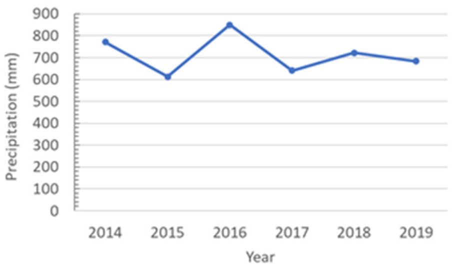

a) Precipitation (2013-2020)

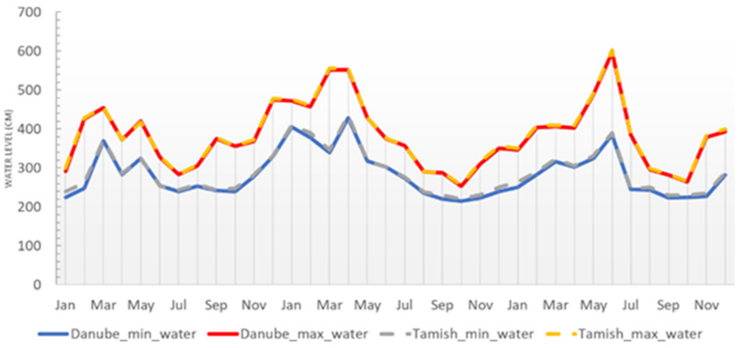

River water levels in 2017 and 2019

Figure S2. Precipitation and river water levels on the territory of South Banat District.

Table S15. The amount of precipitation in the period from 2013 to 2020.

Date	Winter	Spring	Summer	Autumn	Year
Dec 2013 / Nov 2014	↓ 17.6	↑ 329.5	↑ 222.6	↑ 202	↑ 771.7
Dec 2014 / Nov 2015	↑ 151.7	↓ 168	↓ 87.9	↑ 204.6	↓ 612.2
Dec 2015 / Nov 2016	↓ 103.7	↑ 252.6	↑ 280.4	↑ 213	↑ 849.7
Dec 2017 / Nov 2018	↑ 156.9	↓ 162.6	↑ 260	↓ 60.6	↓ 640.1
Dec 2018 / Nov 2019	↑ 167.9	↑ 227.3	↑ 219.5	↓ 107.1	↑ 721.8
Dec 2019 / Nov 2020	↑ 131.5	↓ 109.4	↑ 301.7	↓ 140.4	↓ 683
Maximum	167.9	329.5	301.7	213	849.7
Average	113.7	182.4	212.6	155.1	663.8
Minimum	17.6	35.3	87.9	60.6	180.1

Table S16. Results of a negative binomial regression analysis of the influence of the minimum the Danube river water level fluctuations on the number of registered cases of WNV in humans.

Parameter	B	Std. Error	95% Wald Confidence Interval		Hypothesis Test			Exp(B)	95% Wald Confidence Interval for Exp(B)	
			Lower	Upper	Wald Chi-Square	df	Sig.		Lower	Upper
(Intercept)	6.369	2.2689	1.923	10.816	7.881	1	0.005	583.739	6.839	49827.297
Danube_min	-0.021	0.0089	-0.038	-0.003	5.390	1	0.020	0.980	0.963	0.997
(Scale)	1[a]									
(Negative binomial)	1[a]									

Dependent Variable: WNV_human Model: (Intercept), Danube_min [a]Fixed at the displayed value.

Table S17. Results of a negative binomial regression analysis of the influence of the Danube river average water level fluctuations on the number of registered cases of WNV in humans.

Parameter	B	Std. Error	95% Wald Confidence Interval		Hypothesis Test			Exp(B)	95% Wald Confidence Interval for Exp(B)	
			Lower	Upper	Wald Chi-Square	df	Sig.		Lower	Upper
(Intercept)	6.680	2.1290	2.507	10.853	9.845	1	0.002	796.534	12.274	51692.106
Danube_ave	-0.019	0.0074	-0.034	-0.005	6.841	1	0.009	0.981	0.967	0.995
(Scale)	1[a]									
(Negative binomial)	1[a]									

Dependent Variable: WNV_human Model: (Intercept), Danub_min [a]Fixed at the displayed value.

Table S18. Results of a negative binomial regression analysis of the influence of the Danube river maximum water level fluctuations on the number of registered cases of WNV in humans.

Parameter	B	Std. Error	95% Wald Confidence Interval		Hypothesis Test			Exp(B)	95% Wald Confidence Interval for Exp(B)	
			Lower	Upper	Wald Chi-Square	df	Sig.		Lower	Upper
(Intercept)	5.426	1.7406	2.015	8.838	9.718	1	0.002	227.269	7.498	6888.698
Danub_max	-0.013	0.0051	-0.023	-0.003	6.241	1	0.012	0.987	0.977	0.997
(Scale)	1[a]									

(Negative binomial)	1[a]

Dependent Variable: WNV_human Model: (Intercept), Danub_min [a]Fixed at the displayed value.

Table S19. Results of a negative binomial regression analysis of the influence of the Danube river maximum water level fluctuations and average minimum environmental temperatures on the number of registered WNV positive mosquito positive pools.

Parameter	B	Std. Error	95% Wald Confidence Interval		Hypothesis Test			Exp(B)	95% Wald Confidence Interval for Exp(B)	
			Lower	Upper	Wald Chi-Square	df	Sig.		Lower	Upper
(Intercept)	1.882	2.5673	-3.150	6.914	0.537	1	0.464	6.566	0.043	1005.939
Average_min_t	0.184	0.0916	0.005	0.364	4.051	1	0.044	1.202	1.005	1.439
Danub_max	-0.013	0.0065	-0.026	-0.001	4.231	1	0.040	0.987	0.974	0.999
(Scale)	1[a]									
(Negative binomial)	1.866									

Dependent Variable: WNV-positive_mosquitoe pools
Model: (Intercept), Average_min_t, Danub_max
[a]Fixed at the displayed value

1.4. Prevailing winds direction in South Banat district

The area of South Banat District is flat and characterized by a high frequency of winds. The highest frequency of occurrence is the southeast wind (košava) which occurs at 306 ‰, followed by the northwest wind with 255 ‰, while the lowest frequency of occurrence is the north wind 48 ‰ and the northeast 44 ‰. The prevailing southeast wind most often occurs in autumn 368 ‰, and least often in summer 196 ‰. The highest frequency of silences (Calme) is 143 ‰ in May and the lowest in November 51‰. As for the wind speed, the highest average annual speed for the area has the wind that blows from the east - southeast direction 3.3 m/s, and the lowest south and southwest with a speed of 1.7 m/s. The graphical representation of the dominant winds is shown in Fig. 3. Quantitative data on the distribution and length of the period of blowing of the dominant winds are given in Table 6.

Table S20. Distribution and length of the period of blowing of the prevailing winds in hours of blowing.

Speed km/h	N	NNE	NE	ENE	E	ESE	SE	SSE	S	SSW	SW	WSW	W	WNW	NW	NNW
0	11	8	3	9	0	6	2	0	17	2	5	0	9	3	8	0
1-5	303	252	215	250	105	108	69	52	64	31	42	44	135	181	263	197
6-12	280	282	379	653	546	366	153	45	22	13	19	37	205	734	429	255
13-19	16	24	33	61	201	319	87	7	2	1	2	9	61	424	86	12
20-28	3	6	7	13	37	129	25	1	0	0	0	0	27	216	13	2
29-38	0	1	1	2	4	20	2	0	0	0	0	0	2	36	1	0
39-50	0	0	0	0	0	1	0	0	0	0	0	0	0	1	0	0
51-60	0	0	0	0	0	0	0	0	0	0	0	0	0	0	0	0
>61	0	0	0	0	0	0	0	0	0	0	0	0	0	0	0	0

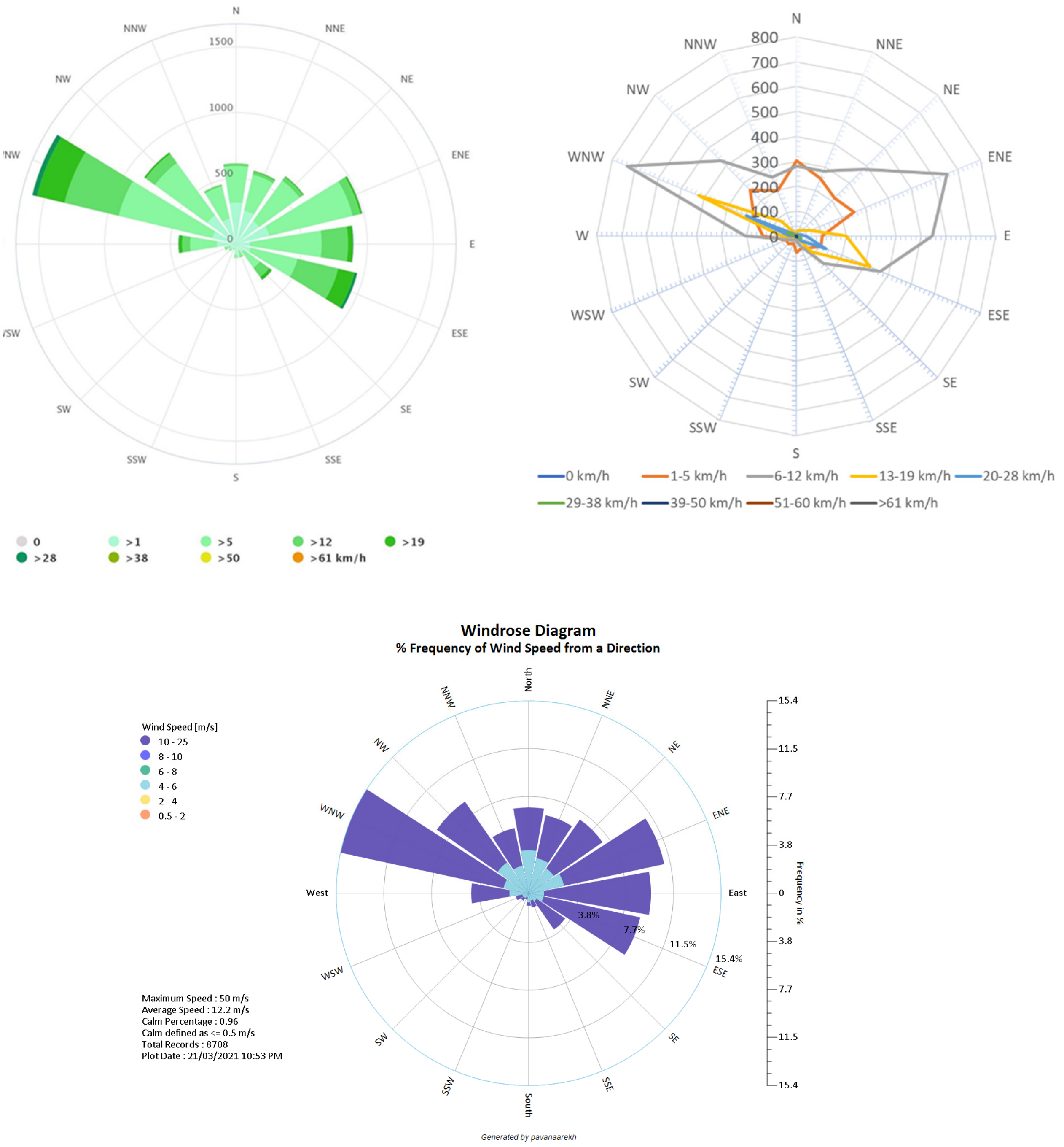

Fig. S3. The wind rose of prevailing winds in South Banat District

2. Geographical distribution of cases WNV in humans and land cover of South Banat district

2.1. Spatial, temporal, and space-time scan statistics

Spatial cluster analysis indicated that the cases of WNV infection were not randomly distributed in space and time between 2017 and 2019. Fig. 7 and 8 show the distribution of cases of WNV infection on the thematic map of the land cover of South Banat District. The map shows the dominant presence of cases in areas rich in water, forests, green vegetation, and inland marshes.

2.1.1. Spatial aggregation

A total of 1 significant and 1 secondary purely spatial cluster were discovered. The most likely and secondary spatial clusters were found in the southwestern part and central part of South Banat District, nearby main water streams (the rivers the Danube, Tamiš, and natural park Ponjavica), including 2 and 9 settlements, respectively. The geographical affiliation of the clusters is as follows (Fig. 4 and 5):

1) Significant cluster: Starčevo, Stari Tamiš,

2) Secondary cluster: Banatsko Novo Selo, Crepaja, Debeljača, Gloganj, Jabuka, Kačarevo, Kovačica, Padina, Sefkerin.

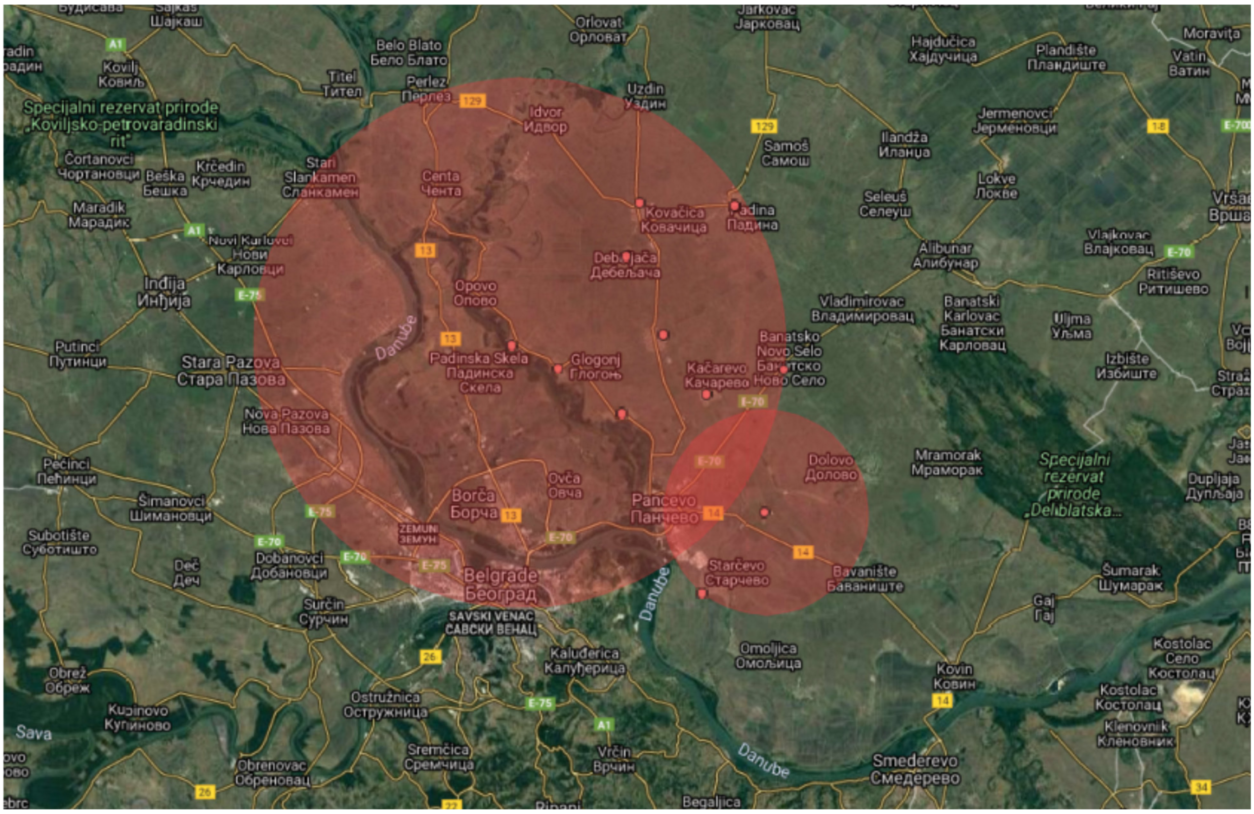

Figure S4. Spatial clusters detected by spatial scan statistic.

2.1.2. Temporal aggregation

A total of 1 purely temporal cluster were discovered (Fig. 6), including 31 settlements.

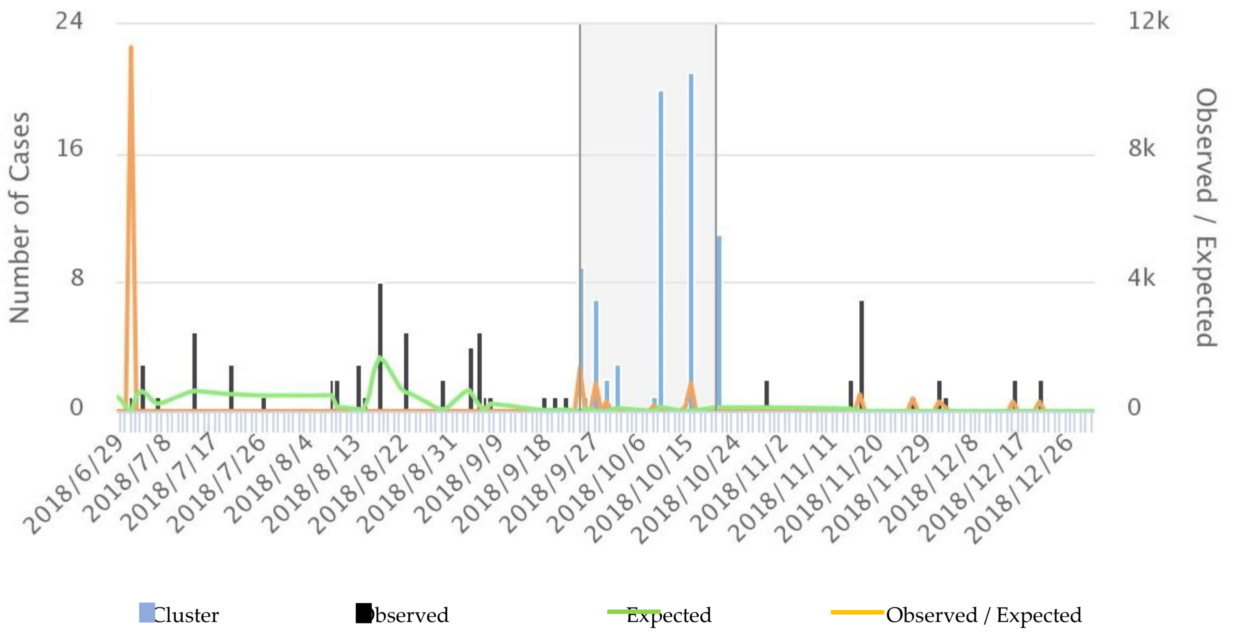

Figure S5. Temporal cluster detected by space-time scan statistic.

2.2. Space-time aggregation

A total of 2 spatio-temporal clusters were discovered, i.e., 1 significant and 1 secondary spatio-temporal cluster were discovered. The most likely and secondary spatio-temporal clusters were found in the southwestern part and central part of South Banat District, nearby main water streams (rivers the Danube, the Tamiš, and natural park Ponjavica), including 12 and 8 settlements, respectively. The geographical affiliation of the clusters is as follows:

1) Significant cluster: Banatski Karlovac, Banatsko Novo Selo, Bavanište, Dolovo, Dupljaja, Gaj, Kovin, Mramorak, Omoljica, Stari Tamiš, Starčevo, Uljma,
2) Secondary cluster: Crepaja, Debeljača, Glogonj, Jabuka, Kačarevo, Kovačica, Padina, Sefkerin.

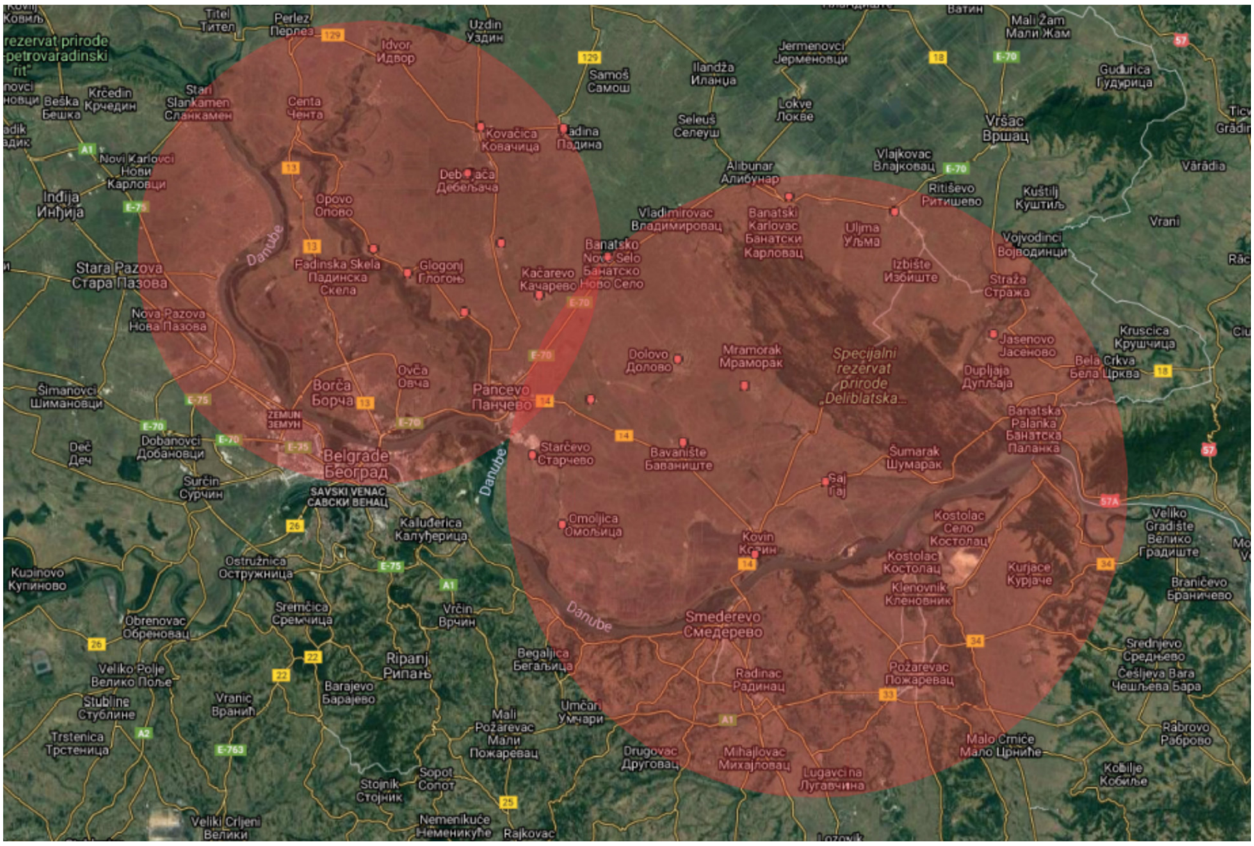

Figure S6. Spatiotemporal clusters detected by space-time scan statistic.

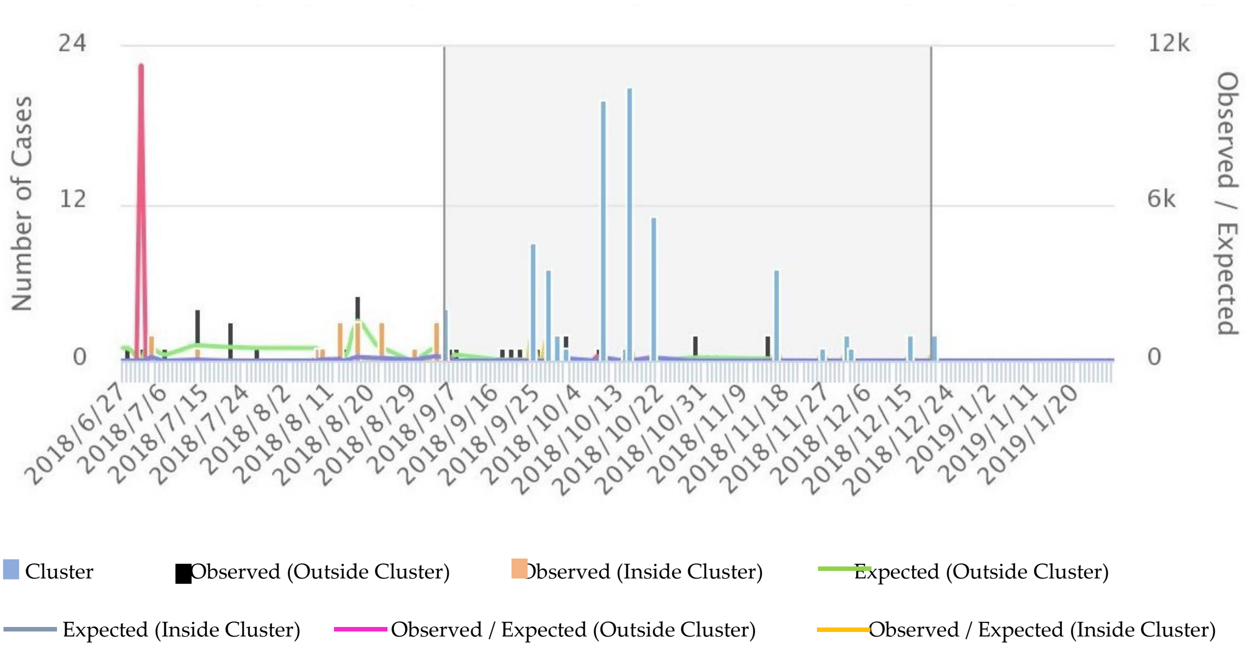

Figure S7. Spatiotemporal cluster detected by space-time scan statistic.

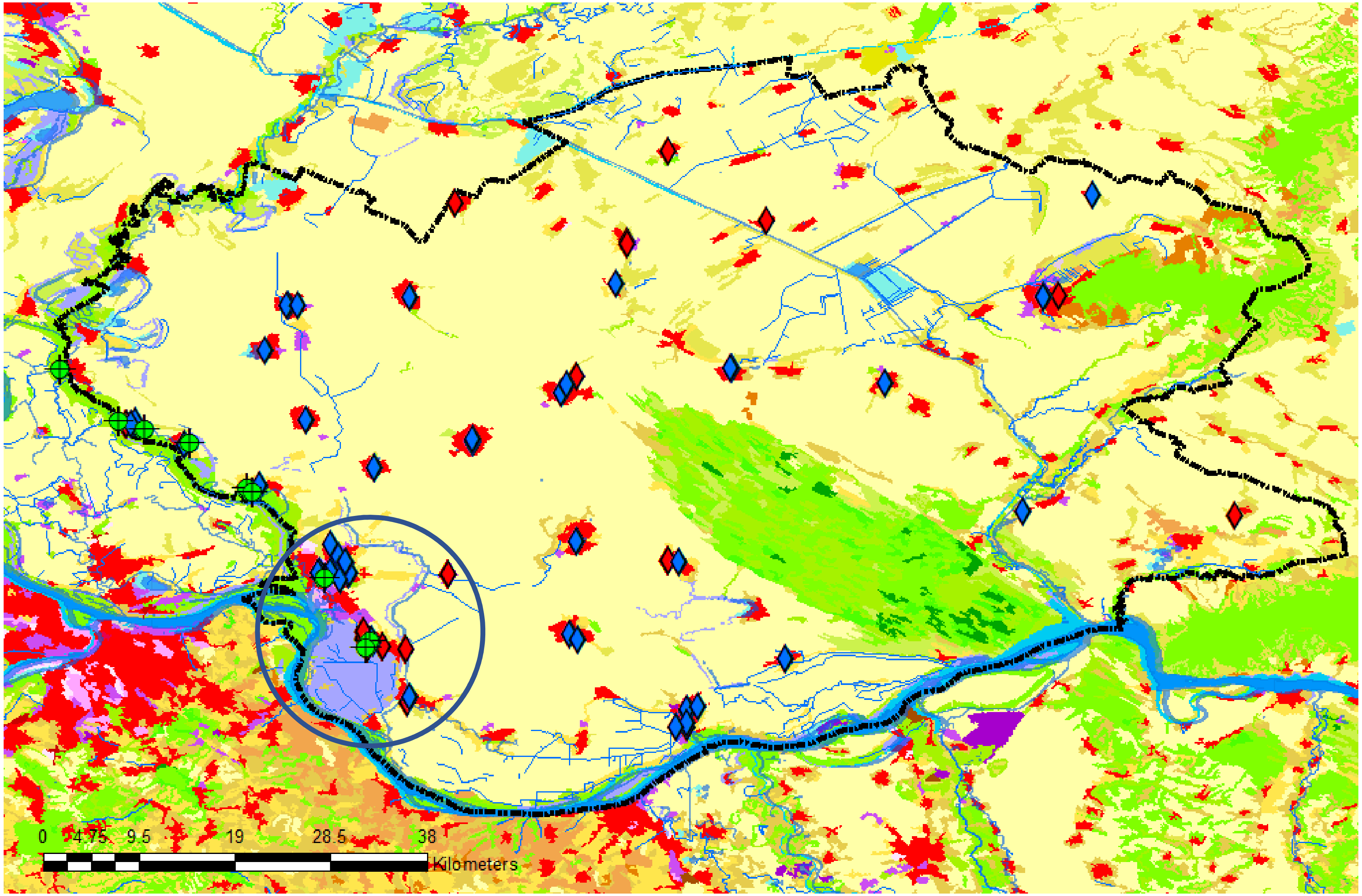

Figure S8. Distribution of registered cases of WNV infection in humans (blue), domestic animals (red), in 2017, 2018, and 2019 and mosquitoes (green) in 2017and 2018 (distribution of cases shown on the thematic map of land cover).

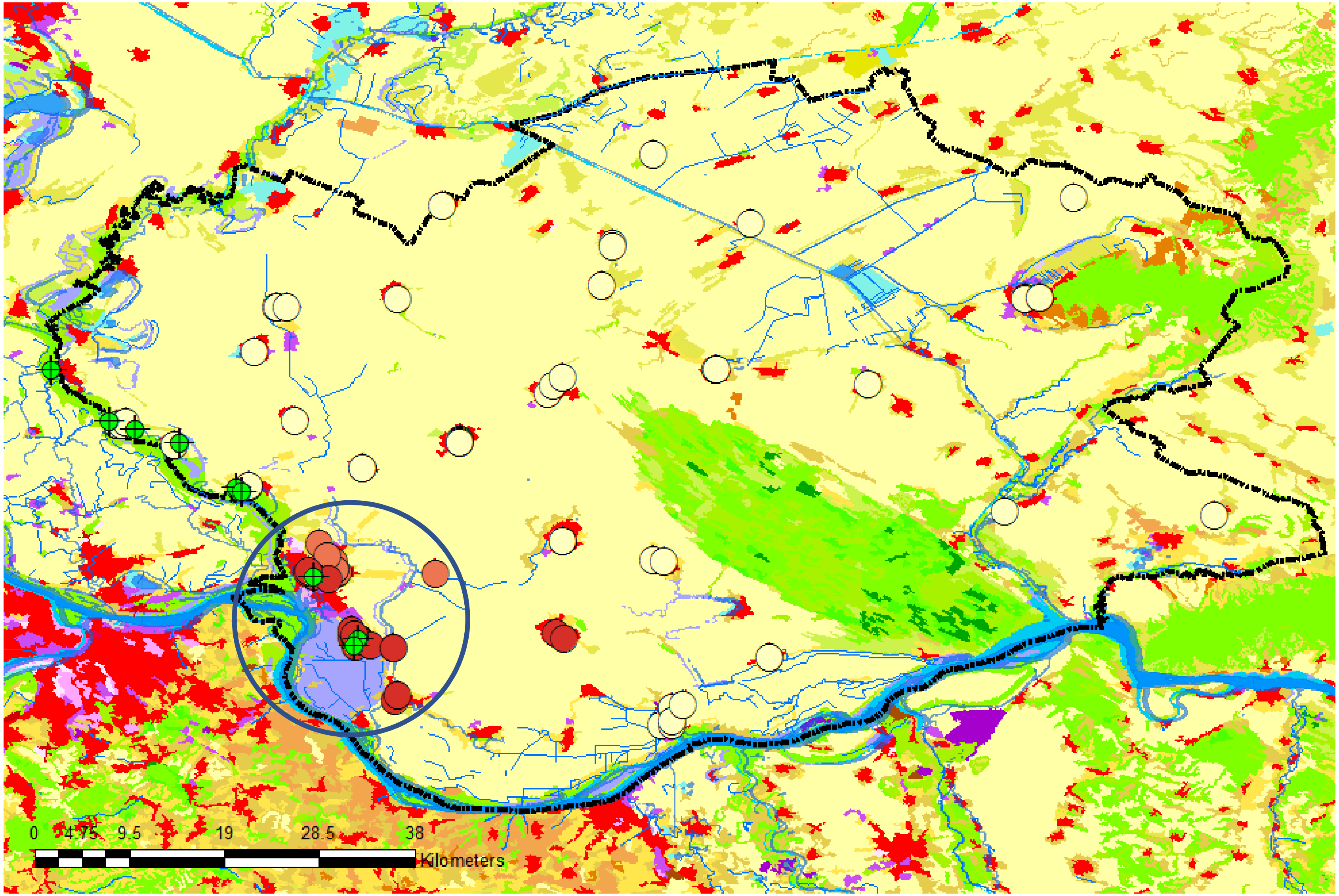

Figure S9. Distribution of hot spots of WNV cases in humans (blue) and domestic animals (red), in 2017, 2018, and 2019 in 2017and 2018 (distribution of cases shown on the thematic map of land cover).

Legend

- WNV_pos_mosquitoes_PRJ
- WNF_human_cases_PRJ
- WNV_animal_spec_PRJ

Land Cover

LABEL3

- Continuous urban fabric
- Discontinuous urban fabric
- Industrial or commercial units
- Road and rail networks and associated land
- Port areas
- Airports
- Mineral extraction sites
- Dump sites
- Construction sites
- Green urban areas
- Sport and leisure facilities
- Non-irrigated arable land
- Permanently irrigated land
- Rice fields
- Vineyards
- Fruit trees and berry plantations
- Olive groves
- Pastures
- Annual crops associated with permanent crops
- Complex cultivation patterns
- Land principally occupied by agriculture, with significant areas of natural vegetation
- Agro-forestry areas
- Broad-leaved forest
- Coniferous forest
- Mixed forest
- Natural grasslands
- Moors and heathland
- Sclerophyllous vegetation
- Transitional woodland-shrub
- Beaches, dunes, sands
- Bare rocks
- Sparsely vegetated areas
- Burnt areas
- Glaciers and perpetual snow
- Inland marshes
- Peat bogs
- Salt marshes
- Salines
- Intertidal flats
- Water courses
- Water bodies
- Coastal lagoons
- Estuaries
- Sea and ocean
- NODATA

3. Epidemiological characteristics of WNV outbreaks in 2017, 2018, and 2019

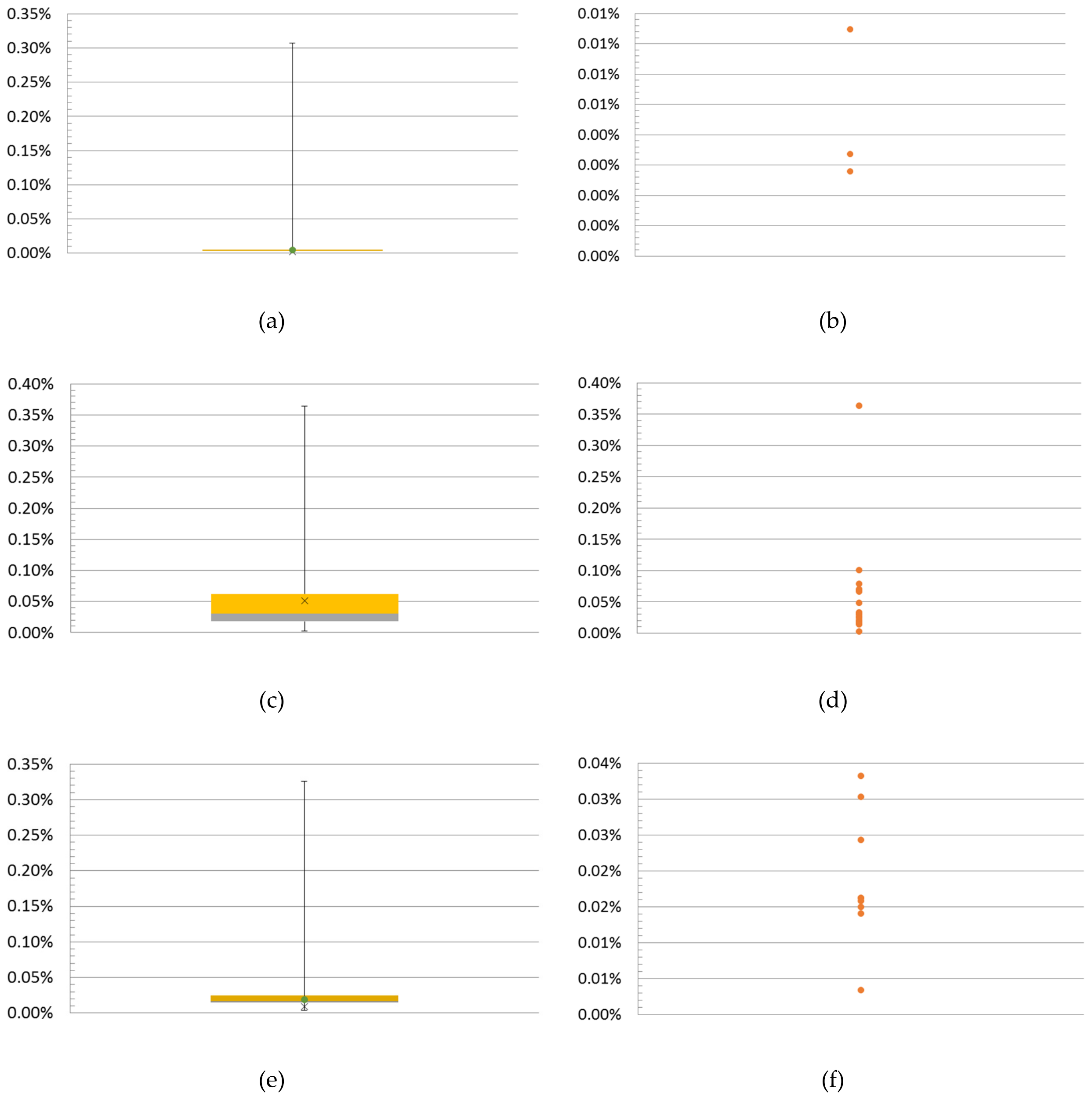

Figure. S10. Appirent prevalence of WNV human cases in stady period. (a) Box plot of WNV prevalence in 2017, (b) Dot plot of WNV prevalence in 2017, (c) Box plot of WNV prevalence in 2018, (b) Dot plot of WNV prevalence in 2018, (e) Box plot of WNV prevalence in 2017, (f) Dot plot of WNV prevalence in 2017
